# Motor learning in golf—a systematic review

**DOI:** 10.3389/fspor.2024.1324615

**Published:** 2024-02-14

**Authors:** Philipp Barzyk, Markus Gruber

**Affiliations:** Department of Sport Science, Human Performance Research Centre, University of Konstanz, Constance, Germany

**Keywords:** skill, motor control, motor learning, performance, practice

## Abstract

Golf is a sport that consists of complex movement skills that need to be executed with utmost precision. Consequently, motor skill learning plays a crucial role in golf, and large numbers of studies address various methods of motor learning. In the present review, we give a systematic overview of randomized controlled trials (RCTs) on motor learning of golf-specific motor skills. Three electronic databases were searched for RCTs looking at the effect of at least one learning method on performance in a golf-specific motor task. We grouped the studies depending on the learning strategies “cognitive training”, “practice scheduling”, “augmented feedback”, “implicit and explicit learning” and “focus of attention”. Fifty-two RCTs met the eligibility criteria and were included in the systematic review. Superior methods within their respective strategies were an external focus of attention and increasing contextual interference, as well as errorless learning. For “cognitive training” and “augmented feedback”, no single method can be considered the most favorable. The overall biggest limitations were the lack of statistical power for more than half of the RCTs, and the fact that most studies of the present review investigated simple putting tasks in novices only. Although we have shown superiority of specific learning methods, transferability of the recommendations that can be derived from simple golf tasks in novices to sport-specific tasks in advanced players still has to be demonstrated and require study designs with the intention to provide practical recommendations for coaches and athletes in the sport of golf.

## 1 Introduction

Movement scientists aim to better understand the fundamental mechanisms of motor learning to deduce guidelines for teachers, coaches, therapists, athletes and patients on how to best learn a sensorimotor skill [for review see (1)]. Various models exist for scientists to study motor learning, with sports being one of the best investigated real-world scenarios in healthy individuals.

In this context, golf can be considered a kind of sport that almost ideally suits the demands for studying specific learning methods. Current research determines performance as a combination of velocity and position of the clubhead at the moment of impact making movement precision key (2, 3). Moreover, the player can freely choose his/her stance and the ball is motionless whilst executing the golf swing, providing a very controllable setting for longitudinal learning experiments. Considering the research to date the performance of a golf shot is easy to assess and rate. As the aim in golf is to move the golf ball from point A (starting position) to point B (final position), quite simply, the distance of the golf ball in its final position relative to point B can be taken as the performance variable (accuracy) of a golf shot. Thus, golf can be considered an ideal sport to study motor learning in a real-world scenario, and it can be expected that the extensively researched topics in motor learning have already been tested within golf-specific tasks.

Despite the advantages of golf for studying the principles of motor learning in a real-world setting, to the best of our knowledge, only a few narrative reviews and book chapters but no systematic review have been published so far (4–9). One publication mainly looked at biomechanics in golf, with one section of the paper dealing with motor learning (6). Additionally, there is one narrative review that exclusively addresses target focused aiming in putting (8). Lee and Schmidt (7) focus primarily on the planning (preparation) and reviewing (evaluation) of the movement. To do so, they discuss the outcomes of studies examining practice schedules (blocked vs. random practice), focus of attention (external vs. internal attentional focus), the duration and stability of gaze before movement execution (quiet-eye effect), and different types of augmented feedback. Wulf and colleagues review studies in golf that are compliant with the OPTIMAL theory of learning (10). They thereby focus on three main factors that are important to optimize learning of new motor tasks (enhanced expectancies, autonomy, and an external focus of attention). Taken together, the aforementioned reviews clearly show that studies on motor learning of golf-specific motor tasks can provide a coherent picture of different learning modalities that are well aligned with the literature on motor learning principles in general (1, 11). Two further theoretical papers are worth mentioning as they give practical recommendations on how to structure motor learning processes in golf based on the “The Challenge Point Framework” (12). This framework provides a theoretical basis for conceptualizing the effects of different exercise conditions in motor learning by relating exercise variables to the individual's performance level and the task difficulty. The Challenge Point Framework for motor learning serves as the basis for both papers in describing the process of learning a golf-related motor skill and is further used to describe how this process can be enhanced. Guadagnoli and Bertram (4) focus on the practice method of contextual interference in relation to the Challenge Point Framework whereas Guadagnoli and Lindquist (5) aim to improve golf performance in general according to the fundamental principles of this framework (4, 5). In these reviews a variety of learning methods have been addressed, however, to date, there is no standardized nomenclature for categorizing the various strategies and underlying methods in motor learning in golf.

In an attempt to structure the approaches that have been studied in motor skill learning in golf, we carefully analyzed the existing reviews and book chapters to establish distinct motor learning strategies that are regularly used in studies so far and discussed in more detail within the golf-specific literature. In this review, we consider motor learning strategies as groups that share common characteristics of practice conditions. The practice forms within these groups are referred to as methods. Noticeable, focus of attention and augmented feedback have been discussed extensively (5–7, 9) and consequently we have used them as two distinct learning strategies within the present review. Another strategy that was characterized in detail was structure of practice (6) or practice scheduling (7). In both cases, learning methods like contextual interference and massed or distributed learning were attributed to this learning strategy. Moreover, different cognitive-related learning methods (e.g., visual illusion or mental practice) have been investigated that we have summarized within the learning strategy “cognitive training”. Besides these four quite distinct learning strategies, awareness of the learned task (implicit/explicit) learning has been discussed on its own (6). The last strategy has some obvious overlap with e.g., focus of attention but can be considered a separate category as some studies specifically look at implicit vs. explicit learning of golf-specific tasks and these studies would not fit into one of the already established strategies. Finally, to structure the present review we will assign the studies that look at different learning methods (e.g., blocked vs. random practice) to one of the five learning strategies (e.g., practice scheduling). This categorization will structure the review by grouping the studies, making it easier for the reader to grasp the main outcomes of the review.

Notwithstanding a great interest within the scientific community on motor learning strategies of golf specific tasks, no systematic review has been published so far that aims to evaluate the studies in this field in a structured way. In the present systematic review, we provide an overview of randomized controlled trials (RCTs) that have investigated the effect of specific motor learning methods on performance of golf-specific motor skills. Following the PICO framework (population, intervention, comparison, and outcome), we tend to answer the question: How do specific motor learning methods affect the performance of participants in a golf-specific task, compared to representative comparison groups that receive no or a different motor learning intervention?

## 2 Methods

### 2.1 Search strategy and selection criteria

**Figure 1:**
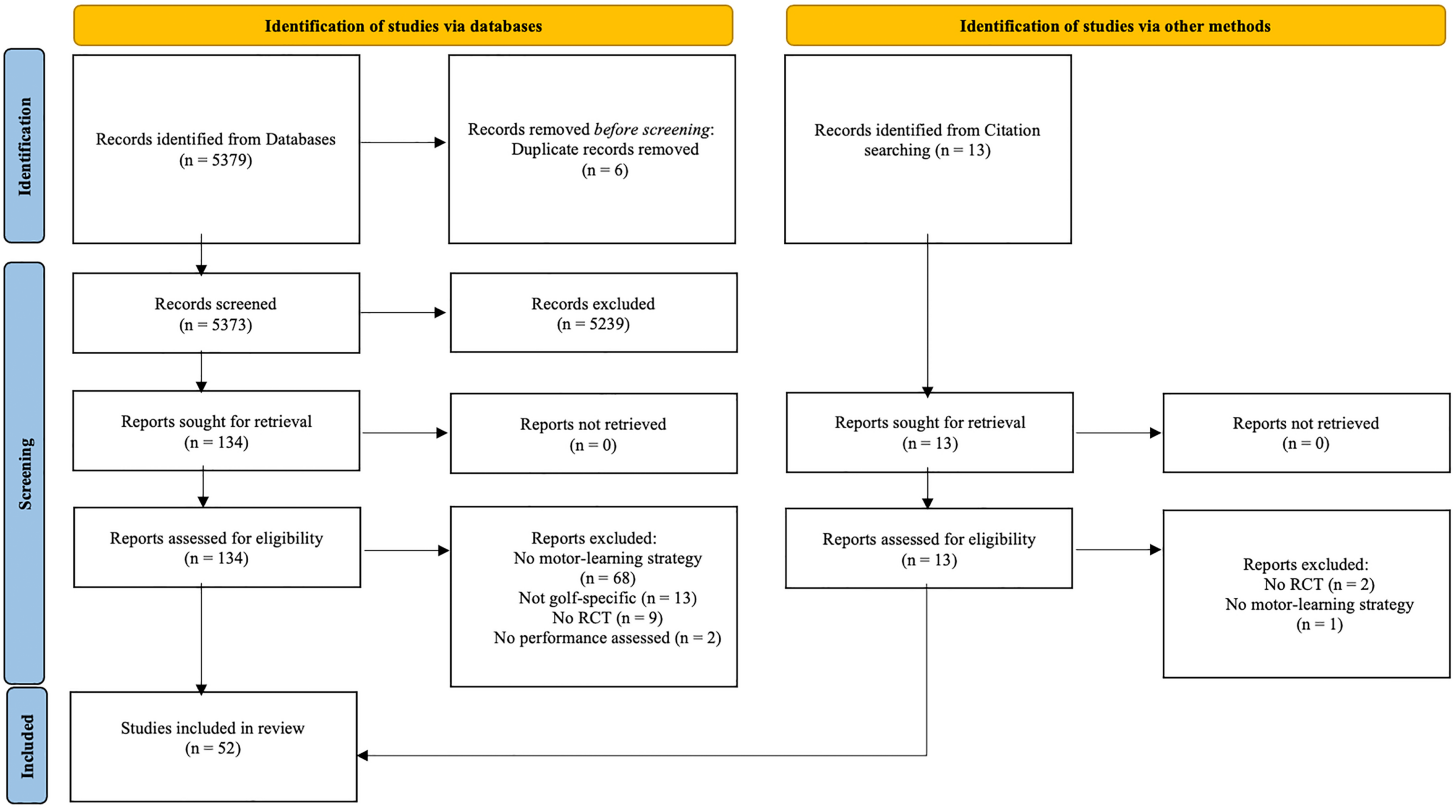
PRISMA flow diagram showing our literature search using various databases, followed by the screening of the publication reference lists as well as a reverse citation search and the final selection process.

Two researchers independently performed an online literature search using three electronic databases (Web of Science, PubMed and SportsDiscus) using publications to date. The papers were selected using the Preferred Reporting Items for Systematic Reviews and Meta-Analyses (PRISMA) guidelines (13). The search of all published studies was performed on the 28th of August 2023. The PICO framework was used to conduct a thorough search strategy. The population was “People learning a golf-specific task”; the interventions studied were “all motor learning strategies”; the comparison was “no learning or a different learning intervention”; the main outcome was “increased performance”. The keywords we derived from this are listed in the appendix. We identified search terms for each PICO element except “comparison” because the diversity of different motor learning strategies makes it difficult to define a fixed comparator. However, following the recommendations of Bobrownicki et al. (14), we considered the relevant comparators when evaluating abstracts and full texts according to the PRISMA guidelines. We conducted the search for “All Fields” but applied the following filters on the results: “Full text, English, peer-reviewed”. First, we screened the titles of the results of this literature search and excluded any publications that did not match our search criteria. Next, we screened each abstract to determine inclusion, and further included papers from the reference lists of the selected publications and computed a reverse citation search using Google Scholar. According to Schmidt et al. (1), motor learning refers to the “processes associated with practice or experience that lead to relatively permanent changes in the capability for skilled movement” (1). In a first step, we therefore excluded non-RCTs and studies without a retention or transfer test to ensure objective comparability (if it was not clear from the abstract whether a study was an RCT, we included or excluded the publication after evaluating the method section of the full text). Finally, we conducted full-text reviews to finalize our decision. Any disagreements that arose during this final phase of screening were resolved through a consensus process involving all members of the review team. The initial search identified a total of 5,379 records. Of these, 6 records were duplicates. Five thousand two hundred thirty-nine records were excluded by title and abstract. One hundred thirty-four reports were retrieved for further screening. Ten reports were added to the selection process during a reverse citation search and reference screening. After a full-text scan, the final research sample included 52 studies (Figure 1). Upon inclusion in the review, we extracted the key information from each article.

### 2.2 Data extraction

We extracted the following information from each publication: (1) author(s) and year of publication; (2) methodological details [e.g., study design, participant characteristics (e.g., age, sex, handicap, duration, outcome measures, and motor learning strategies); and (3) main findings (mean and SD) with respect to the effectiveness of the intervention (e.g., improved motor performance) (Tables 1–5)]. To better distinguish between the participants, we categorized them into different skill levels based on their handicap: “Novice” for no handicap (HCP), “low skill level” for HCP 36–54, “mid skill level” for HCP 10–36, and “high skill level” for HCP under 10. To further differentiate and compare the studies within groups, we divided them into groups focusing on mental training through the use of visual occlusion or mental imaging (“cognitive training”); altering the structure of practice, including distribution of practice and contextual interferences (“practice scheduling”); different forms of augmented feedback such as video-feedback or the effects of knowledge of results (“augmented feedback”); and finally, differences due to varying attention during practice, such as implicit and explicit learning strategies; or different attentional foci during execution (“implicit and explicit learning”, “focus of attention”). We defined these groups as motor learning strategies, as they share common features of practice conditions. Furthermore, to distinguish the specific forms of practice within these groups, we categorized them into groups based on learning method. For example, a trial in which participants were instructed to set their attentional focus during acquisition of a motor skill was, by definition, assigned to the “focus of attention” learning strategy. The group that practiced with an external focus of attention was assigned to the “external focus of attention” learning method, and the group that practiced with an internal focus of attention was consequently assigned to the “internal focus of attention” learning method. Risk of bias (RoB) was assessed using the RoB 2 tool for RCTs, which follows the Cochrane Handbook for Systematic Reviews of Interventions (66). Overall, the ratings for the RoB assessment were predominantly “some concerns”, mostly due to the lack of availability of information. Only four studies were rated as having a high RoB (See Appendix for detailed assessment). To be able to go into more detail on the relevance of the individual results, we extracted each study's effect size. Using G*Power (version (3.1.9.6), we then carried out a *post-hoc* power analysis for the main outcomes of all studies in the present review. For such analyses, a statistical power *>*.8 is desirable, as it is considered to be the threshold for relevant statistical effects (67).

**Table 1:** Studies examining performance changes in golf-specific motor skills as a result of cognitive training.

Authors	Skill/skill level	Number of participants	Age (mean)	Sex	Timing of retention test	Characteristics of the learning intervention	Main results
Bahmani et al. (15)	Putting/novice	30	10 SD = 0.41	M	Two days after the last learning session	50 trials: group A: visual illusion (larger hole), group B: visual illusion (smaller hole)	Significant higher increase in performance for group A compared to group B at retention
Beilock et al. (16)	Putting/novice	126	19.35 SD = 1.68	M = 34 F = 92	N. R.	50 trials (2 m putt): Group A: low frequency positive imagery, group B: high frequency positive imagery, group C: low frequency suppression, group D: high frequency suppression, group E: low frequency suppression-replacement, group F: high frequency suppression-replacement, group G: control	Significant increase in performance for groups A, B, C, E an G. Group D performed significantly worse than groups A, B, C and E. Group F performed significantly worse than groups A and C
Chauvel et al. (17)	Putting/novice	36	21.7 SD = 1.25	M = 16 F = 20	One day after the last learning session	50 trials (2 m putt): Group A: visual illusion (larger hole), group B: visual illusion (smaller hole)	Significant higher increase in performance for group A compared to group B at retention
Frank et al. (18)	Putting/novice	52	24.67	M = 18 F = 34	72-h after the last learning session	60 trials: Group A: mental practice, group B: physical practice, group C: mental-physical practice, group D: control	No differences between the groups at retention
Kim et al. (19)	Putting/novice	40	25 SD = 4.12	M = 18 F = 22	One day (post-test) and 2 days (retention) after the last learning session	Three days (60 trials/day): Group A: action observation training, group B: motor imagery training, group C: physical practice, group D: control (no practice)	Significant increase in performance at post-test and retention test for group A and group C
Lewthwaite et al. (20)	Putting/novice	24	20.6 SD = 2.76	M = 16 F = 8	One day after the last learning session	60 trials (4 m putts): Group A: choice, group B: yoked	Significant higher increase in performance for group A compared to group B at retention
Meacci and Pastore (21)	Putting/novice	80	19 SD = N. R	M = 52 F = 28	Five weeks after the last learning session	Ten weeks (25 trials/3× per week): Group A: visual contact and imagery, group B: nonvisual contact and imagery, group C: visual contact, group D: control	Significant increase in performance for all three intervention groups at retention. No difference between group A and B, but both groups performed significantly better than group C
Palmer et al. (22)	Putting/novice	34	24.6 SD = 5.2	M = 12 F = 22	One day after the last learning session (retention and transfer)	50 trials (1.5 m putt): Group A: visual illusion (larger hole), group B: visual illusion (smaller hole)	Significant higher increase in performance for group A compared to group B at retention and transfer
Taylor and Shaw (23)	Putting/novice—high-skilled	51	20.2/18.8	M = 21/25 F = 4/1	N. R.	24 practice trials and 16 test-trials (4.5 m putt): Group A: positive imagery, group B: negative imagery, group C: control	Significant higher decrease in performance for group B compared to groups A and C after post-test. No difference in groups A and C
Ziv et al. (24)	Putting/novice	45	N. R.	M	Two days after the last learning session (retention and transfer)	50 trials (1.5 m putt): Group A: visual illusion (larger hole), group B: visual illusion (smaller hole), group C: control	No significant difference at retention. Significant higher increase in performance for group A compared to groups B and C at transfer
Ziv et al. (25)	Putting/novice	45	23.9 SD = 2.7	M = 13 F = 32	Two days after the last learning session (retention and transfer)	50 trials (2 m putt): Group A: visual illusion (larger hole), group B: visual illusion (smaller hole), group C: control	No significant difference at retention. Significant higher increase in performance for group A compared to groups B and C at transfer
Ziv et al. (26)	Putting/novice	76	23.3 SD = 2.97	M = 21 F = 55	48-h after the last learning session	50 trials: Group A: visual aid behind hole, group B: visual aid in front of hole, group C: visual aid around hole, group D: visual aid placed according to participant	No difference in Absolute error between the groups at retention. Variable error was significantly higher for group B compared to groups C and D

N. R., not reported; significant (higher) increase in performance, compared to pre-test.

**Table 2:** Studies examining performance changes in golf-specific motor skills as a result of practice scheduling.

Authors	Skill/skill level	Number of participants	Age (mean)	Sex	Timing of retention test	Characteristics of the learning intervention	Main results
Chua et al. (27)	Putting/novice	36	26.1 SD = 8.45	M = 17 F = 19	Two days after the last learning session (retention and transfer)	60 trials (20× from three distances): Group A: blocked, group B: random	No difference at retention. Significant higher increase in performance for group B compared to group A at transfer
Dail and Christina (28)	Putting/novice	90	22 SD = N. R	M = 25 F = 65	One, seven and 28 days after the last learning session (only 15 participants per day)	Group A: 240 trials (one day) massed practice, group B: 60 trials (4 days) distributed practice	Significant higher increase in performance for group B compared to group A
Fazeli et al. (29)	Putting/novice	30	27.4 SD = 4.6	M	One week after the last learning session	Group A: 180 trials for 6 days random practice, group B: 180 trials for 6 days blocked practice, group C: no-practice control	Significant higher increase in performance for group A compared to group B. Group C performance significantly worse than groups A and B at retention
Goodwin and Meeuwsen (30)	Putting/novice	30	26 SD = 8.0	F	One day after the last learning session (retention and transfer)	198 trials: group A: random, group B: blocked—random, group C: blocked	No difference in the three groups at retention and 1.67-or 3.19 m-transfer. Significant higher increase in performance for group A and B compared to group C at 6.23 m- transfer
Porter and Magill (31)	Putting/novice	60	N. R.	M = 18 F = 42	N. R. (retention and transfer test)	81 trials: group A: Blocked, group B: increasing, group C: random	Significant higher increase in performance for group B compared to both other groups at retention. No difference between groups A and C. Significant higher increase in performance for group B compared to group C at transfer. No difference to group A or between groups A and C
Schmidt et al. (32)	Putting/novice	42	24 SD = 3.6	M = 30 F = 12	One and 2 weeks after the last learning session (retention and transfer)	288 trials: group A: contextual interference, group B: differential learning, group C: differential learning plus putter variation, group D: control	Significant increase in performance for all groups pre -post. Significant decrease in performance for group D at retention test 1. No differences between groups on retention test 2. Comparable results for transfer test 1 and 3. Significant decrease in performance for group D at transfer

N. R., not reported; significant (higher) increase in performance, compared to pre-test.

**Table 3:** Studies examining performance changes in golf-specific motor skills as a result of different forms of augmented feedback.

Authors	Skill/skill level	Number of participants	Age (mean)	Sex	Timing of retention test	Characteristics of the learning intervention	Main results
An et al. (33)	Putting/novice	30	22.4 SD = 1.41	M = 20 F = 10	One day after the last learning session	60 trials (2 m putts): group A: choice, group B: control	Significant higher increase in performance for group A compared to group B at retention
Biénkiewicz et al. (15)	Putting/novice	30	19 SD = 2.4	M = 24 F = 6	Two weeks after the last learning session (retention and transfer)	120 trials: group A: auditory guidance, group B: visual guidance, group C: control	No differences between groups at retention or transfer test
Butki and Hoffman (34)	Putting/novice	78	N. R.	M = 48 F = 30	Five minutes and 34 h after the last learning session	96 trials: group A: knowledge of result-deprived 50%, group B: knowledge of result-deprived 100%, group C: continuous knowledge of result	Increase in performance for groups A and B at delayed retention trials compared to Group C
Chiviacowsky et al. (35)	Putting/novice	28	23 SD = 6.71	M = 14 F = 14	One day after the last learning session	50 trials: group A: positive temporal-comparative feedback, group B: control	Significant higher increase in performance for group A compared to group B at retention
de Souza Nunes et al. (36)	Putting/novice	40	69 SD = 2.95	M = 18 F = 22	Immediate and 24 h after the last learning session (retention and transfer)	120 trials (2 days): Group A: “self” or group B: “yoked” knowledge of performance	No differences between groups at retention or transfer test
Guadagnoli et al. (37)	7 iron full swing/mid—low	30	N. R. (range: 29–50)	N. R.	Two and 14 days after the last learning session	90-min session (4 days): group A: video, group B: verbal, group C: self-guided	Error distance: no differences between groups. Total distance: Significant increase in performance for group A only at retention two. No change for both other groups
Jalalvand et al. (38)	Putting/novice	60	21 SD = 1.59	M = 32 F = 28	One day after the last learning session (retention, single-task transfer and dual-task transfer)	80 trials: group A: dual-factor gradual self-control group, group B: self-controlled task difficulty group, group C: self- controlled feedback group, group D: dual-factor yoked group	Significant higher increase in performance for group A at retention compared to groups B, C and D. No difference between groups A and B at both transfer tests, but significantly better performance compared to groups C and D
Pourbehbahani et al. (39)	Putting/novice	40	26.10 SD = 5.56	M = 20 F = 20	One day (retention) and 2 weeks (follow-up) after the last learning session	216 trials (3 blocks, 2 m putts): group A: neurofeedback/self-controlled practice, group B: neurofeedback/yoked practice, group C: sham/self-controlled practice, group D: sham/yoked practice	All groups increased performance at retention. groups A and B upheld that effect at follow-up
Post et al. (40)	9 iron chipping/novice	44	22 SD = 1.3	M = 6 F = 38	One day after the last learning session (retention and transfer)	60 trials (6 blocks, 15 m): Group A: self-control, group B: yoked	No differences between groups at retention. Significant higher increase in performance for group A at transfer compared to group B
Ring et al. (41)	Putting/mid skill	24	22	M	One day after the last learning session	180 min of putting: Group A: neurofeedback, group B: yoked control	Significant increase in performance for all groups, without differences between them
Smith et al. (42)	PW chipping/novice	48	22 SD = 3.6	M = 24 F = 24	One day after the last learning session	50 trials: group A: knowledge of result 0%, group B: knowledge of result 5%, group C: knowledge of result 10%	Significant higher increase in performance for group B at retention compared to group A

N. R., not reported; significant (higher) increase in performance, compared to pre-test.

**Table 4:** Studies examining performance changes in golf-specific motor skills as a result of implicit and explicit learning.

Authors	Skill/skill level	Number of participants	Age (mean)	Sex	Timing of retention test	Characteristics of the learning intervention	Main results
Chauvel et al. (43)	Putting/novice	48 young 48 older	Young: 23.5 SD = 3.3, Older: 65.0 SD = 3.7	Young: M = 24 F = 24, Older: M = 25 F = 23	Immediately after the last learning session	160 trials: Group A: errorful, group B: errorless, group C: errorful dual-task, group D: errorless dual-task	Higher performance for group A compared to Group C, but no difference to Groups B and D regardless of age
Hardy et al. (44)	Putting/novice	32	21.23 SD = N. R.	M = 16 F = 16	One day after the last learning session	100 trials: group A: implicit learning, group B: implicit dual task, group C: explicit learning, group D: control	Significant increase in performance for all groups, without differences between them
Lam et al. (45)	Putting/novice	36	21 SD = 2.03	M = 22 F = 14	Same day as the last learning session (retention and transfer)	600 trials: group A: errorless from short to long distance, group B: errorful from long to short distance	Significant higher increase in performance for group A compared to group B at retention and transfer
Masters (46)	Putting/novice	40	27.22	N. R.	One day after the last learning session	400 trials (4 days): Group A: implicit learning, group B: explicit learning, group C: implicit control, group D: stressed control, group E: non-stressed control	Increase in performance for groups A, C and D. Decrease in performance for groups B and E
Maxwell et al. (47)	Putting/novice	27	23 SD = 2.17	N. R.	72 h after the last learning session	3,000 trials (5 days): group A: explicit learning, group B: implicit learning (plus implicit control)	No difference between groups at retention (objectively better performance of group B)
Maxwell et al. (48)	Putting/novice	29	21 SD = 2.4	N. R.	N. R. [retention test and two transfer tests (secondary and novel task)]	400 trials (8 blocks×8 distances): Group A: errorless from short -long, group B: errorful from long-short, group C: random	Significant higher increase in performance for group A compared to both other group at retention. No difference between group B and C
Moore et al. (49)	Putting/novice	40	19.55 SD = 1.65	N. R.	One day after the last learning session (retention and transfer)	320 trials: group A: quiet-eye, group B: technical training	Significant increase in performance for group A compared to group B at retention and transfer
Poolton et al. (50)	Putting/novice	35	21.1 SD = 1.48	M = 11 F = 24	One day after the last learning session (retention and transfer)	400 trials: group A: explicit learning, group B: implicit-explicit learning	No differences between the groups at retention. Significant increase in performance for group B compared to group A at transfer
Vine et al. (51)	Putting/high skilled	22	20.95 SD = 2.66	M	N. R.	20 trials: group A: quiet-eye, group B: control	Significant increase in performance for group A compared to group B at retention and transfer
Vine et al. (52)	Putting/novice	45	21.22 SD = 4.41	N. R.	One day after the last learning session (retention and transfer)	320 trials: group A: quiet eye, group B: analogy learning, group C: explicit learning	Significant increase in performance for group A compared to groups B and C at retention and transfer. No differences between groups B and C
Zhu et al. (53)	Putting/novice	18	22	N. R.	One day after the last learning session (retention and transfer)	300 trials: group A: errorless learning, group B: errorful learning	Significant increase in performance for group A compared to group B at both retention and transfer

N. R., not reported; significant (higher) increase in performance, compared to pre-test.

**Table 5:** Studies examining performance changes in golf-specific motor skills as a result of different foci of attention.

Authors	Skill/skill level	Number of participants	Age (mean)	Sex	Timing of retention test	Characteristics of the learning intervention	Main results
Aiken and Becker (54)	GW chipping/novice	79	19.28 SD = 2.31	M = 27 F = 52	One day after the last learning session	80 trials (8 blocks): Group A: internal focus of attention, group B: external focus of attention, group C: internal to external switching group	No difference for group B compared to groups A and C at retention. Significantly higher increase in performance for group C compared to group A
An et al. (55)	7 iron/low	24	27.3 SD = 2.05	M	Three days after the last learning session	30 trials (3 blocks): Group A: internal focus of attention, group B: external focus of attention, group C: control	Significant higher increase in performance for group A compared to groups B and C at retention
Bell and Hardy (56)	PW chipping/high	33	37.06 SD = 17.84	M	N. R.	100 trials (4 blocks, 20 m): group A: internal focus of attention, group B: proximal external focus of attention, group C: distal external focus of attention	Significant higher increase in performance for group C compared to groups A and B
Brocken et al. (57)	Putting/novice	60	9 SD = 0.45, 12 SD = 0.43	M = 26 F = 34	One day after the last learning session	80 trials: group A&C: external focus of attention instructions, group B&D: internal focus of attention instructions	Significant higher increase in performance for group A and C at retention compared to group B and D
Christina and Alpenfels (58)	6 iron/mid	45	65 SD = 7.79	M	One day after the last learning session	36 trials (3 blocks): Group A: internal focus of attention, group B: external focus of attention, Group C: control	Significant higher increase in performance for group B compared to group C at retention
Christina and Alpenfels (58)	Driver/mid	39	64 SD = 15	M	One day after the last learning session	36 trials (3 blocks): Group A: internal focus of attention, group B: external focus of attention, Group C: control	No significant difference between groups A, B and C
Land et al. (59)	Putting/high	30	48 SD = 14.37	M	N. R.	40 trials: group A: external focus of attention, group B: irrelevant focus of attention, group C: control	Significant higher increase in performance for group A at retention compared to groups B and C. No difference between groups B and C
Lawrence et al. (60)	Putting/novice	29	22 SD = 4.3	N. R.	Shortly after the last learning session [retention with low/high anxiety (LA/HA)]	200 trials: group A: internal focus of attention, group B: external focus of attention, group C: control	Significant increase in performance from LA to HA for group B and no change in performance for group A. Significant decrease in performance from LA to HA for group C. Mean error significantly decreased for all groups. Group C had a significantly greater error compared to group B
Munzert et al. (61)	Putting/novice	30	N. R.	M = 9 F = 21	One day after the last learning session (switching between attention foci)	120 trials: group A: internal focus of attention, group B: external focus of attention	Better performance for group B at retention compared to group A. Increase in absolute error for group B and decrease in absolute error for group A when receiving the opposite instructions
Perkins-Ceccato et al. (62)	9 iron pitch/high and low	20	N. R.	M = 16 F = 4	N. R.	80 trials: group A: switching from internal to external focus of attention, group B: switching from external to internal focus of attention	Highly skilled golfers performed better with an external focus of attention, whereas low-skilled golfers performed better with an internal focus of attention
Poolton et al. (63)	Putting/novice	30	24 SD = 5.94	M = 7 F = 23	Immediately after the last learning session (without instructions or with secondary task)	300 trials: group A: internal focus of attention, group B: external focus of attention	No differences between groups at retention. Significant decrease in performance for group A at transfer
Wulf et al. (64)	9 iron pitch/novice	22	N. R. (range: 21–29)	M = 13 F = 9	One day after the last learning session	80 trials: group A: internal focus of attention, group B: external focus of attention	Significant higher increase in performance for group B compared to group A at retention
Wulf et al. (65)	9 iron pitch/novice	30	N. R.	N. R.	One day after the last learning session	60 trials: group A: internal focus of attention, group B: external focus of attention: group C: control	Significant higher increase in performance for group B at retention compared to groups A and C. No difference between groups A and C
Wulf et al. (65)	9 iron pitch/high	6	N. R.	N. R.	One day after the last learning session	60 trials: group A: internal focus of attention, group B: external focus of attention: group C: control	Significant higher increase in performance for group B at retention compared to groups A and C. No difference between groups A and C

N. R., not reported; significant (higher) increase in performance, compared to pre-test.

## 3 Results

### 3.1 Cognitive training

As the term “cognitive training” is rather broad, researchers have differentiated between different forms (Table 1). In general, cognitive training has been proposed as a method to facilitate motor learning, especially in the elderly and neurologically impaired population (68, 69). Cognitive training is the training of motor skills in which the learner does not participate in physical activity. Examples include visual occlusion, perceptual-cognitive training and mental imaging. However, its general efficacy is still under debate, as some studies only show short-term enhancements of motor learning [for review see (70)] whereas others argue the current state of research does not show clear benefits of cognitive training (71, 72). We identified 12 studies that used some form of cognitive training to study motor learning in golf.

We found four RCTs that investigated motor imagery and/or action observation in comparison to traditional practice during a putting task (16, 18, 19, 23). Motor imagery is a method where people imagine themselves carrying out a motor task in order to enhance performance (73). The observation of a movement (action observation, for example through a video) has been previously shown to be beneficial in motor learning [for review see (74)]. Both methods are often investigated simultaneously, particularly within the scope of research related to sports, due to the comparable involvement of neurons (75). Beilock and colleagues (16) assigned 126 participants into seven groups (low frequency positive imagery, high frequency positive imagery, low frequency suppression, high frequency suppression, low frequency suppression-replacement, high frequency suppression-replacement no-imagery control). The groups tasked with suppression were instructed to avoid imagining undershooting or overshooting the target. Whereas the suppression-replacement groups were instructed to imagine the ball sitting atop the target upon imagining either overshooting or undershooting. They performed 20 practice trials and 30 test trials with their respective imagery task. All groups except for high frequency suppression and high frequency suppression-replacement improved their performance. Frank and colleagues (18) assigned 52 novice golfers to one of four groups (imagery, physical, combined imagery and physical, and no practice). Each intervention group completed sixty putting trials using their respective training method. No differences were found between the groups at retention. In the study from Kim and colleagues (19), 40 participants were randomly assigned to one of four groups (action observation, motor imagery, physical practice, control). They performed 180 putts over the course of 3 days plus a retention test 1 and 2 days after the intervention. All three intervention groups improved significantly compared to the control group at both post-test and retention, with no differences between the three intervention groups. Taylor and Shaw (23) explored the effects of positive and negative outcome imagery on a putting task. They divided 25 novices and 26 experienced participants (high skill level) into three groups (positive imagery, negative imagery, control). In the positive imagery condition, participants were instructed to envision a perfect shot prior to each putt. In the negative imagery condition, participants envisioned one of four negative images before every putt. These images included the ball landing short right, hard right, short left, or hard left of the hole. Each participant performed 24 practice putts and 16 putts with their respective imagery task. The negative imagery groups performed significantly worse than the positive and control groups, with the novices being affected significantly more than the high skilled players. There was no difference between the positive imagery and control group.

Another study investigated the effects of occluded vision and imagery on putting accuracy (21). Eighty novice golfers were divided into three different intervention groups and one control group. One intervention group performed the putting task with visual contact and imagery, another one with nonvisual contact (eyes closed until the ball was retrieved by an experimenter) and imagery, and a third group solely with visual contact. Participants trained three times per week over 10 weeks, and then performed a retention test 5 weeks after the acquisition period. All three intervention groups increased putting performance significantly at retention without any significant differences between groups.

Another study looked at the effects of visual aids on putting performance (26). Seventy-six participants, divided into four groups (visual aid behind hole, visual aid in front of hole, visual aid around hole, visual aid placed according to participant) performed 50 putting trials plus retention. No differences in absolute error (deviation from hole) were found between the groups, but variable error (putting consistency) was significantly higher in the group with the aid placed in front of the hole compared to those with the aid placed behind the hole or those who could choose the placement. Similar to the group that was able to choose the placement of the visual aid, one study focused solely on the effects of autonomy during a golf putting task (20). Autonomy-supportive conditions (i.e., choosing a ball-color, placing a visual aid) can enhance learners' overall confidence and self-efficacy for specific tasks. The process of decision making and the expectation of having the opportunity to make decisions is additionally linked to increased activity in areas of the brain that deal with reward processing. Such increased brain activity may result in stronger interests in learning a task, as well as greater emotional involvement (20). Twenty-four participants were divided into either a choice group, able to select their color of golf balls, or a yoked group, which were provided with pre-selected golf balls of the same color as a participant matched to them in the choice group. Results of a retention test showed significantly greater putting accuracy for the choice group compared with the yoked group.

Previous research showed that increased confidence can lead to a higher task-performance (76) and based on that, different groups have investigated the effects of visual illusions and how this could influence performance. Witt et al. (77) facilitated the “Ebbinghaus-illusion” to show that the perception of a bigger target can lead to improved task-performance (77). Several studies followed this approach, by investigating the effects of visual illusions on putting performance (15, 17, 22, 24, 25). In these studies, participants performed putts whilst either perceiving a larger or a smaller hole. Interestingly, the groups that putted towards the perceived larger hole showed a significantly better performance at retention whilst putting without any visual illusion in all studies except for Ziv et al. (24, 25) where those groups only showed increased performance in a transfer test which involved putting from a previously untrained distance.

### 3.2 Practice scheduling

In this section of the review, we focus on studies that examined the effectiveness of exercise planning (1). Practice scheduling includes six studies that investigated either “distribution of practice” or “contextual interference” (Table 2).

Distributed practice uses time intervals (usually hours or days) between training sessions, while massed practice consists of fewer or shorter intervals between trials during training sessions (78). Studies on “distribution of practice” investigate how practicing multiple task variations compared to practicing only one task variation as measured in retention and transfer tests. Dail and Christina (28) divided 90 novice golfers into two groups. Participants of the first group performed each time 60 putts on 4 days (distributed practice) and participants of the second group performed 240 putts on the same day (massed practice). The distributed practice group performed significantly better during retention compared to the massed practice group.

Contextual interference is a learning strategy in which interference during practice has a positive effect on motor skill learning. While random practice may temporarily decrease performance during acquisition, it ultimately can improve learning as measured by retention and transfer test results (79). Five studies investigated the effects of contextual interference on putting performance. These included four studies which directly compared blocked and random practice during a putting task (27, 29–31) and one study that added an additional differential learning group (32). During blocked practice, the participants completed all trials of a given task before moving on to the next one. Therefore, the focus lied on the repetition of one motor skill without interruption. On the other hand, during random practice the given task was varied across the session to practice a different task during every new attempt (80). Goodwin and Meeuwsen (30), Porter and Magill (31) and Fazeli et al. (29) each included three groups of novice golfers with either a blocked, random, and no-practice or combined strategy, whereas Chua et al. (27) only included a random and a blocked group. In the study by Porter and Magill, 60 participants were divided into three groups (blocked, increasing, random). All groups performed 27 putting trials from three different distances. The blocked group practiced 27 consecutive trials at one location, before moving on to the next. The random group practiced all trials randomly, with the restriction that no more than two trials were putted from the same location on consecutive trials. The group with increasing trials started with blocked trials transitioning to a randomized order. In the study by Goodwin and Meeuwsen, 30 participants were assigned to either a random, blocked-random, or blocked group and performed 66 trials, also from three different distances. Fazeli and colleagues included 30 participants, divided into three groups (blocked, random, no-practice), with the practice groups performing 180 putting trials per day for 6 days. In the study by Chua and colleagues, 36 participants were divided into two groups and completed 60 putting trials from different distances. Except for (29) the other three studies included a transfer test that required the participants to perform ten trials each from novel distances. Neither Goodwin and Meeuwsen (30) nor Chua et al. (27) showed significant differences at retention, but for both studies significantly less absolute error for the random (as well as the blocked-random group in the study by Goodwin and Meeuwsen) groups compared to the blocked groups during the transfer test were reported. Porter and Magill (31) found that the combined condition led to significantly better performance in comparison to the blocked and random conditions during the retention test, and also to better performance compared to the random condition during a transfer test. Fazeli et al. (29) showed increased performance for both blocked and random groups compared to the control group, with the random group performing significantly better than the blocked one. In the last study, 42 novice golfers, divided into one contextual interference group (increasing contextual interference from blocked to random order), two differential-learning groups (different putter for each trial and/or movement variations) and a control group, completed eight sessions of putting practice over 4 weeks, followed by two retention tests and a transfer test. The intervention groups performed significantly better at the first retention test compared to the control group, without statistical differences between them (32).

### 3.3 Augmented feedback

As defined by Sigrist et al. (81), augmented feedback (or extrinsic feedback) refers to information that necessarily requires an external source to contextualize it. Eleven studies were specifically designed to investigate the effects of different forms of augmented feedback on golf performance (Table 3). We included one study studying the effects of guidance (visual vs. auditory modalities) on putting performance (82). We consider guidance to be a subcategory of augmented feedback, as it relies on external instructions to convey information to the learner while physically assisting them through the task to be learned (1). Kümmel et al. (83) already have demonstrated the effects of haptic guidance, in their study by a robotic device, on learning specific hand kinematics during the backswing (83). Bieńkiewicz et al. (82) assigned 30 novice golfers to either an auditory guidance group, a visual guidance group, or a control group. Each participant performed 120 putts from three distances, plus a ten-trial retention test 2 weeks after the acquisition phase. In addition, they performed a transfer test from two novel distances directly after the acquisition phase. No significant differences were found between groups, neither for retention nor transfer.

Two other studies investigated performance due to differences in knowledge of results (KR) (34, 42). KR refers to augmented feedback that provides information about the movement outcome (e.g., where the golf ball lands), which is distinct from knowledge of performance (KP), which relates to information about the quality of the movement execution itself (e.g., the extent of hip extension or the extent of shoulder rotation) (11). Smith et al. (42) divided 48 novice golfers into three groups (0%, 5% and 10% Bandwidth KR). The 0% group received feedback after every trial, and the two others only if the error was outside their bandwidth (5% or 10%). They further differentiated between providing traditional KR (outcome information) and transitional information (corrective feedback towards errors in movement patterns). Each group performed fifty chips (10 m distance) during the acquisition phase, plus a retention test 1 day later. There was only an increase in performance for the 10% Group at retention when receiving transitional information. Butki and Hoffman (34) compared the effects of continuous KR with two KR-deprived groups. One KR-deprived group (50/50 group) received visual feedback of the ball path on 50% of the trials, whilst being deprived of the putting outcome on the other 50%. The second KR-deprived group (0/50 group) did not receive any visual feedback of the ball path at all but received feedback on the final ball location for 50% of the trials. Twenty-eight participants per group performed 96 putting trials, plus two retention tests. Even though the continuous KR group performed better during acquisition, the KR-deprived groups performed better during retention, with an advantage for the 0/50 group.

In another study, Chiviacowsky and colleagues (35) compared putting performance between positive temporal-comparative feedback and a control group (35). Positive temporal-comparative feedback compares performance results for one individual across practice trials, practice blocks, or practice sessions. Participants in the positive temporal-comparative feedback group were falsely informed of an increase in performance compared to the previous blocks, with the intention to increase their expectations for improved performance in the future. This form of feedback was chosen by the authors of the study, as previous studies suggested that motivation can play a role in how feedback is received and how it affects the process of motor learning (84–86). Twenty-eight novice golfers performed 50 putts whilst receiving verbal feedback on their performance. The positive temporal-comparative group was further provided with false feedback that suggested an increase in performance. At retention, the positive temporal-comparative group performed significantly better than the control group.

Pourbehbahani et al. (39) explored the effects of neurofeedback [sensorimotor rhythm (SMR) neurofeedback] and self-controlled practice (choosing the color of the practice balls) on novice golfers. They divided 40 participants into four groups (neurofeedback/self-controlled practice, neurofeedback/yoked practice, sham/self-controlled practice and sham/yoked practice). The participants performed 216 putting trials and a retention test (1 day later) as well as a follow-up test (2 weeks later). Neurofeedback and self-controlled practice both independently led to performance enhancement at retention. At follow-up testing only neurofeedback upheld these effects. Similarly, one other study looked at the effects of neurofeedback in mid-skilled golfer in comparison to a yoked control group (41). Twenty-four participants received either true or false neurofeedback [Electroencephalography (EEG)-signal as audio tone] for three one-hour sessions over separate days before putting performance was assessed in a retention test. Both groups improved their performance, however no differences were found between the two groups.

One study focused on the effects of video feedback on golf performance using a seven iron (37). Thirty amateur golfers (HCP between −7 and −16) were split into three groups (video, verbal, and self-guided feedback). The study consisted of four ninety-minute acquisition sessions with a break of one day in between the sessions. The study also included two retention tests, the first one 2 days after the end of the last acquisition phase, and the second 2 weeks later. No differences in “error distance” were observed between groups; however, for the second retention task, the video feedback group showed significant increases in performance (“total distance”) in comparison to both the verbal and the self-guided group. Similar to Guadagnoli et al. (37), four other studies investigated self-controlled feedback. Post et al. (40) as well as de Souza Nunes (36) and An et al. (33) compared self-controlled feedback with a control condition (33) or a yoked condition, where participants received feedback according to the self-controlled group (36, 40). An et al. (33), as well as Post et al. (40), were able to show better performance for the self-guided groups, whereas de Souza Nunes (36) reported no differences between groups. The inconsistent results could be due to the differences in age groups between the studies. An et al. (33) and Post et al. (40) included young adults, whereas de Souza Nunes (36) included elderly people. The fourth study (38) included 60 novice golfers who were divided into four groups based on two factors: task difficulty control and feedback control. The “dual-factor gradual self-control group” could select both the task difficulty, by choosing a predetermined distance, and the timing of verbal feedback. The “self-controlled task difficulty group” could choose the task difficulty, but the feedback was yoked. The “self-controlled feedback group” could choose the feedback, but the task difficulty was yoked. Lastly, the “dual-factor yoked group” received both yoked task difficulty and feedback. Each group performed eighty trials of a putting task whilst receiving 100% KP (feedback on errors in movement execution) provided by an instructor during the first 40 trials, and either dual-factor gradual self-control or self-controlled feedback during the second forty trials. After one day they performed retention, transfer, and dual-task transfer tests. The dual-factor gradual self-control group performed significantly better than the other groups. However, for both transfer tests, there was only a difference between the dual-factor gradual self-control group and the self-controlled feedback group.

### 3.4 Implicit and explicit learning

We found 11 studies examining either the effects of implicit vs. explicit or different forms of implicit learning on putting performance (Table 4). Explicit motor learning focuses specifically on parts of the whole movement in order to learn the entire task (for example, different parts of the swing pattern), whereas implicit motor learning happens without awareness of the learned task and how it is performed (46).

Three studies investigated the effects of implicit and explicit learning on golf putting (44, 46, 47). In the first study, 40 novice golfers were divided in five groups (implicit, explicit, implicit control, stressed control, and non-stressed control). They performed 400 putting trials over four sessions and a hundred putts during retention. Both implicit learning groups as well as the non-stressed control group improved their performance over time, whereas the explicit and stressed control groups decreased in performance (46). The second study involved 32 novice golfers divided into four learning groups (implicit, implicit dual-task, explicit and control). They completed 100 putting trials and a retention test. All four groups improved their performance significantly, with no differences between them (44). In the third study, 27 novice golfers were divided into an explicit and implicit group, as well as an implicit control group. They performed three thousand putting trials over 5 days, as well as a retention test after 72 h (47). All groups significantly improved their performance over time without any significant differences between the groups.

A method commonly used in implicit learning research is the acquisition of a novel skill with reduced or increased number of errors. Four studies (43, 45, 48, 53) investigating the effects or errorful and errorless learning during a golf putting task. Errorless learning is a learning technique that reduces errors during the learning process, especially in the initial stages of learning (87). In contrast, errorful learning facilitates or promotes errors during the learning process. The studies compared situations where the error in putting accuracy was either minimized or maximized throughout the learning session. The errorless groups started putting from the shortest distance and finished at the longest distance, whereas the errorful groups started at the longest distance and finished at the shortest distance. Chauvel et al. (43) divided 48 younger and older novice golfers into four groups (errorful, errorless, errorful dual-task, errorless dual-task). They performed 160 putts and a retention test. No significant differences were found between the learning groups at retention. However, both errorless groups performed significantly better than the errorful groups at transfer. Lam et al. (45) included 29 novice golfers, Maxwell et al. (48) 36 and Zhu et al. (53) 18, all divided them into two groups (errorful vs. errorless). The participants performed 400 (45), 600 (48) and 300 trials (53) of a putting task over eight different distances (range from 25 cm to 200 cm in 25 cm steps). All three studies consistently reported the errorless groups to have better putting accuracy in both retention and transfer tests compared to the errorful and random groups. In addition, Maxwell et al. (48) introduced a secondary task, using tone counting during putting. They reported robust performance in the errorless group, whereas the performance of the errorful and random groups decreased significantly during the dual task test. One study compared the effects of explicit and implicit-explicit learning using errorless learning only (50). Thirty-five novice golfers were divided into an explicit and an implicit-explicit learning group. Each group completed 400 putting trials as well as a retention and transfer test. There was no significant difference between the groups at retention, but the implicit-explicit group outperformed the explicit group at transfer.

Another method used in the context of implicit learning is “quiet-eye” (QE). In QE the participants are instructed to focus on a specific target (e.g., the golf ball) for 2–3 s, prior to initiation a movement (7). We found three studies that looked at the effects of QE-training compared to either a control group (51), an explicit learning group (49), or an analogy learning group and an explicit group (52). Participants were instructed to focus their gaze on the back of the ball before beginning their stroke and to continue gazing at the same spot after striking the ball. In the study by Vine et al. (51) 22 high-skilled golfers, divided into two groups (QE, control) performed twenty golf putts using their respective learning method as well as a retention and transfer test. The intervention group performed significantly better at both retention and transfer. Moore et al. (49) divided 40 novice golfers into a QE-group and an explicit learning group (participants received technical putting instructions). Similar to Vine et al. (51), a retention test and a transfer test were conducted. The QE-group performed significantly better compared to the explicit learning group in both tests. In the last study, Vine et al. (52) divided 45 novice golfers into a QE-group, an explicit learning group as well as another implicit learning group (analogy learning), where participants were asked to “keep [their] body still like a grandfather clock and use [their] arms the same way that the pendulum of the clock operates” (52). Each group performed 320 putting trials, followed by a retention and transfer test. The QE-group performed significantly better than the other two groups at retention and transfer. There was no significant difference between the explicit and analogy learning groups.

### 3.5 Focus of attention

Twelve studies examined the effects of internal and external foci of attention during golf practice (Table 5). An internal focus of attention directs attention during movement execution toward the movements of the involved bodily parts, whereas an external focus of attention directs attention toward the movement outcomes (88). Similar to the majority of studies we have discussed so far, putting was used in 5 out of 12 studies that examined the effect of attentional foci (57, 59–61, 63). Brocken et al. (57), Land et al. (59) and Munzert et al. (61) were able to show the advantages of an external focus of attention (even though Land and colleagues only compared it to an irrelevant focus of attention and a control group). Lawrence et al. (60) showed mixed results, whereas Poolton et al. (63) did not see any differences between the two methods, except for a decrease in performance for an internal focus of attention at transfer testing.

Lawrence et al. (60) examined the different foci of attention together with induced anxiety. They hereby distinguished between the number of successful putts and the mean radial error as outcome parameters. Twenty-nine novice golfers performed two hundred putts in one of three groups (internal focus of attention, external focus of attention, control), plus 25 putts with low anxiety and 25 putts with high anxiety shortly after the acquisition phase. Results showed an increase in performance for all groups for both outcome parameters. However, when comparing the performance of putts with high vs. low anxiety, the authors showed a significant decrease in the number of successful putts for the control group, a significant increase for the external focus group, and no change for the internal focus group. For mean radial error, the only significant difference was an increase in performance in the external focus group when compared to the control group.

One study focused on the effects of different attentional foci on seven iron swing performance (55). Twenty-four participants were divided into three groups: an internal focus group, an external focus group and a control group. They performed 90 full seven iron swings. Three days later, they performed a retention, and showed significantly larger carry distances for the external focus group in comparison to all other groups. Christina and Alpenfels (58) performed similar experiments for six iron and driver performance [(58); experiments one & two]. However, they either demonstrated only a difference between the external focus group and control (experiment one) or no difference between the groups at all (experiment two).

Two studies focused on chipping performance using a pitching wedge (56) or gap wedge (54). Bell and Hardy (56) divided 33 participants into three groups. The internal focus of attention group was instructed to focus on their arms during the swing, whereas the proximal external focus of attention group focused on the position of the clubface throughout the swing and the distal external focus of attention group on the flight of the ball. All participants performed 100 chips towards a 20-m target. Both external focus groups performed significantly better than the internal focus group, with the distal external focus group performing even better than the proximal external focus group (56). Similarly, Aiken and Becker (54) divided 79 novice golfers into three groups (internal focus, external focus and internal/external switching focus). Initially, they found no differences between groups at retention. However, when removing participants who reported low adherence to the attentional focus instruction, they found significantly better performance for the switching group compared to the internal group. There was no difference between the switching or internal group compared to the external group (54).

The last three studies examined the effects of different foci of attention on nine iron pitching performance (62, 64, 65). Wulf and colleagues had 6–30 subjects practice pitching 60–80 times over 15 m. One day later, they conducted a retention test and demonstrated significantly better performance for an external focus of attention during acquisition in both novices [(64, 65) experiment one] and highly skilled golfers [(65) experiment two]. Similarly, Perkins–Ceccato and colleagues (62) included ten novices and ten highly skilled golfers in their study. Each group performed the pitch ten times from four different distances. The participants changed their focus of attention after half of the swings (either from internal to external or vice versa). The highly skilled golfers performed significantly better after instructions with an external focus of attention, while the novices performed significantly better after instructions with an internal focus of attention.

## 4 Discussion

To the best of our knowledge, the present review on motor learning in golf is the first review that systematically collects motor learning studies for a specific sport. The studies in golf cover the most-discussed motor learning strategies in general, such as: augmented feedback, cognitive training, practice scheduling, implicit and explicit learning and focus of attention. The results on the learning of golf-like and golf-specific skills are well in line with results from other motor learning or motor relearning studies. It should be mentioned that almost half of the studies we found were underpowered, explaining at least part of the non-significant results. Moreover, most studies are designed to reveal the effects of motor learning methods in one single learning strategy. Further, most interventions were simple golf tasks, like short putts, and the participants in the studies were most often golf novices, which together makes a transfer to both practice and the actual game of golf rather difficult. In the present review, we identified 52 RCTs that specifically looked at learning of golf-specific motor skills. We were able to categorize the studies into five groups, each of the groups looking at one specific learning strategy (focus of attention, augmented feedback, practice scheduling, cognitive training, implicit and explicit learning, see Table 6.

**Table 6:** Results of a *post-hoc* power analysis to determine the statistical evidence level for each study and the different motor learning strategies.

Motor learning strategy	Number of papers	Main outcome	Mean effect size	Mean stat. power
Focus of attention	12	External focus >internal focus	.54 ± .20	.64 ± .29
Cognitive training	12	Inconclusive	.44 ± .16	.63 ± .29
Augmented feedback	11	Augmented feedback >control	.61 ± .23	.89 ± .14
Implicit vs. explicit learning	11	Inconclusive	.45 ± .18	.60 ± .25
Practice scheduling	6	Distributed practice >massed practice	.50 ± .34	.70 ± .22

Power and effect sizes are calculated for each study individually and averaged for the respective motor learning strategy. Mean effect size is given as Cohen's f. An effect size of 0.1 reflects a small, 0.25 a medium and 0.4 a large effect. A mean statistical power of 0.5 indicates the chance to detect the real effect with a chance of 50%.

We were able to show that the basic motor learning strategies are present in golf studies and found the vast majority of studies to be focused either on a varying focus of attention or different types of augmented feedback.

### 4.1 Focus of attention

There is considerable evidence and a general consensus in the literature that an external focus of attention facilitates motor performance and motor learning [for meta-analysis and review see (88, 89)]. The results of the present review (Table 5) support this result within the learning of putting (57, 59–61, 63) and chipping (54, 56). These results are well in line with several sport-related studies, for example, in darts (90), basketball (91, 92), and gymnastics (93). Studies in other research areas, such as (neuro-)rehabilitation, which primarily aim at motor skill relearning, have also been able to confirm the positive effects of an external over an internal focus of attention [for review see (94)]. When we look at golf studies, we can already observe better performance in the groups that practice with an external focus of attention during the first block of trials when compared to the internal focus or control groups (65). This has been attributed to the fact that by turning away the learner's attention from his own body or body parts (which would be an internal focus of attention), conscious thinking does not impair automatized motor programs to any further extent, which ensures a timely and precise movement execution [for review see (95)]. It is beneficial that better performance with an external focus of attention is also accompanied by better learning, making it an easy decision to give privilege to an external focus of attention while implementing this learning strategy into practice. For a more detailed discussion on attentional foci and the learning of golf-specific motor skills, we direct the reader's attention to Lee and Schmidt [(7), p. 9ff] and Chua et al. [(88), p. 618ff].

However, although most studies show that an external focus of attention is advantageous over no attentional instruction or an instruction that directs the learner's attention internally, recent reviews have raised doubts about the reliability of these results. By re-analyzing the data on previously published meta-analyses with Bayesian meta-analysis methods, McKay and colleagues (96) reported moderate to strong evidence of publication bias for all previous analyses and clear evidence of heterogeneity in each analysis which considerably weakens the hitherto postulated clear evidence for a strong effect of the focus of attention as an effective strategy in motor learning (96). Besides more statistical confounding factors in the literature so far, Herrebrøden (97) discussed the concept of “task-relevant information”, as a mechanisms that can be used to explain at least some of the heterogeneity between previous studies and questions the strong conclusions on the superiority of an external focus of attention during motor learning in terms of its content (97).

In conclusion, there is still evidence pointing towards a primacy of an external over an internal focus of attention for learning golf specific tasks, but this evidence has been questioned recently with some valid arguments. Future studies in this field need to ensure comparability of instructions and take into account statistical bias as well as the concept of “task-relevant information” in order to test and validate the superiority of an external over an internal focus of attention during motor learning.

### 4.2 Augmented feedback

Eleven studies focused on different forms of augmented feedback. Augmented feedback has been studied extensively, and it is widely accepted that it can be used as a key element to facilitate motor skill learning [for review see (81)]. The findings of golf studies in the field of augmented feedback are highly variable, with many studies not being able to show an advantage of one form of augmented feedback over another, nor over the classic form of training without augmented feedback, or even against no training at all (36, 38, 40, 41, 82). Despite the varying and occasionally conflicting results, most studies suggest that augmented feedback can be beneficial in motor learning. The effects of positive temporal-comparative feedback in golf are in line with the results of previous studies which reported better performance for positive than negative temporal-comparative feedback during a coincident timing task (84) or target throwing (85). Furthermore, the results on verbal feedback, self-guided feedback, and video feedback are supported by a recent review that examined studies comparing video and verbal feedback (98). The overall positive effects of augmented feedback fit well with the results of other studies on motor skills that found this learning strategy to be effective (99). Augmented feedback is also utilized outside of a sporting context. For example, it is often used in physiotherapy rehabilitation to promote motor learning for injury prevention. The results shown in the golf studies are hereby consistent with studies that examined the effects of augmented feedback on the rehabilitation process of lower extremity musculoskeletal dysfunction (100).

It should be noted that there are many different forms of augmented feedback (verbal, auditory, visual, etc.) as well as different ways of presenting augmented feedback that influence motor learning and that their effectiveness in improving motor learning seems to interact with the skill level of the participants (novice vs. advanced) and the timing (concurrent vs. delayed) [for reviews see (81, 101)]. To investigate motor learning in golf Biénkiewicz and colleagues (82) explored the effects of auditory and visual guidance, while Pourbehbahani and colleagues (39) as well as Ring and colleagues (41) focused on the effects of neurofeedback. An et al. (33) provided tactile feedback to one of their learning groups, and Guadagnoli et al. (37) as well as Post et al. (40) provided video feedback. Most studies used verbal or visual feedback for at least one of their intervention groups. Verbal feedback was used in studies by Guadagnoli et al. (37), Jalalvand et al. (38), Chiviacowsky et al. (35), and de Souza Nunes et al. (36). Visual feedback was used in studies by Biénkiewicz et al. (82), Butki and Hoffmann (34), An et al. (33), and Smith et al. (42). Many studies have shown that multimodal feedback in particular, i.e., the combination of two or more forms of feedback, can help to improve performance and can be superior to a single form of feedback [for reviews see (81, 102)]. However, multimodal augmented feedback has not yet been investigated in golf so far. Future research should therefore look at which combinations of augmented feedback could be most beneficial for learning processes in order to make practical suggestions for coaches, therapists, and athletes.

### 4.3 Practice scheduling

Looking at the studies investigating practice scheduling (Table 2), the overall positive effect of increasing contextual interference (29, 31) as well as distributed or varying practice (28, 32), is in line with previous research. Similar results have already been reported for other sports such as basketball (103) and also for other fields, like neurorehabilitation (104). However, some studies investigating contextual interference were only able to show positive effects of random over blocked practice during transfer tests (27, 30). Contextual interference (blocked vs. random practice) has already been discussed extensively in the review by Lee and Schmidt (7). In their review, they emphasize the specificity of motor learning and recommend practices that best simulate the specific demands of the play on the course. Each golf shot on the course is unique and is not a repetition of the previous one. As motor learning is highly specific to the task (105, 106) it is necessary for the practice to reflect the core features of the play on the course. While the results presented here are consistent with the majority of previous literature, there might be a dependency on skill level as Porter and Magill (31) showed benefits of increasing contextual interference the longer a person trained. This is further discussed in another paper by Porter and Beckerman (107), who use the Challenge Point Framework to discuss how learners are constantly challenged when contextual interference increases during practice to a level that leads to improved performance. To propose some practical suggestions, we refer to Guadagnoli and Bertram (4), who give application-oriented examples of contextual interference in relation to the Challenge Point Framework. The authors describe common problems golfers of different skill levels face when practicing and offer solutions to these problems. They therefore propose, the better you are, the fewer balls you should hit in succession and/or with the same club. On the other hand, adding too much difficulty and variation to the training process for a less skilled player could be counterproductive as it adds too much “challenge”.

### 4.4 Implicit and explicit learning

Three studies looking at errorful vs. errorless learning consistently reported a benefit of errorless over errorful learning, especially when introducing a secondary task, with medium to large effect sizes (Table 7). Novice players seem to benefit from errorless learning which fits the theory behind the Challenge Point Framework, as it suggests that, to optimize learning, the level of difficulty should be matched to the level of the learner's skill. Thus, this theory calls into question the generalizability of such results, as the effects could be completely reversed for advanced golfers or professional athletes. Nevertheless, the results of the three studies are well in line with other studies investigating the effects of errorful and errorless practice, for example with throwing activities (108), memory learning in children (109) and also for relearning motor skills in neurorehabilitation (110). Lam et al. (45), Maxwell et al. (48) as well as Zhu et al. (53) argue that reducing errors in acquisition leads to implicit learning processes and is therefore superior to an errorful approach. Their rationale is that implicit learning processes are dominated by procedural forms of motor control without relying on the working memory. Explicit learning processes, on the other hand, are dominated by declarative forms of motor control that do rely on the working memory. However, the benefits of implicit learning over explicit learning are still inconclusive. As an alternative explanation psychological factors, especially reward, may promote the superiority of errorless vs. errorful learning. Studies investigating the effects of intrinsic rewards have found that people are more inclined to engage in activities that are intrinsically rewarding. Reward is linked to increased self-efficacy and positive emotions, both important prerequisites for optimal learning processes [for review see (111)].

**Table 7:** Effect size and power.

Authors	Group size	Group age	Group gender	Stat. power	Effect size (f)
Studies examining performance changes in golf-specific motor skills as result of cognitive training					
Bahmani et al. (15)	*N* = 30	10.66 SD = 0.41	M	0.99	0.68
Beilock (16)	*N* = 126	19.35 SD = 1.68	M = 34 F = 92	0.90	0.28
Chauvel et al. (17)	*N* = 36	21.7 SD = 1.25	M = 16 F = 20	0.97	0.52
Frank et al. (18)	*N* = 52	24.67	M = 18 F = 34	0.44	0.28
Kim et al. (19)	*N* = 40	25.2 SD = 4.12	M = 18 F = 22	0.43	0.50
Lewthwaite et al. (20)	*N* = 24	20.6 SD = 2.76	M = 16 F = 8	0.64	0.50
Meacci and Pastore (21)	*N* = 80	19.3 SD 17–23	M = 52 F = 28	0.99	0.77
Palmer et al. (22)	*N* = 34	24.6 SD = 5.2	M = 12 F = 22	0.30	0.38
Taylor and Shaw (23)	*N* = 51	20.2/18.8	M = 21/4 F = 25/1	0.59	0.31
Ziv et al. (24)	*N* = 45	N. R.	M	0.28	0.39
Ziv et al. (25)	*N* = 45	23.9 SD = 2.7	M = 13 F = 32	0.28	0.38
Ziv et al. (26)	*N* = 76	23.3 SD = 2.97	M = 21 F = 55	0.53	0.38
Studies examining performance changes in golf-specific motor skills as result of practice scheduling					
Chua et al. (27)	*N* = 36	26.1 SD = 8.45	M = 17 F = 19	0.81	0.49
Dail and Christina (28)	*N* = 90	22.3 SD = n.r.	M = 25 F = 65	0.89	0.28
Fazeli et al. (29)	*N* = 30	27.4 SD = 4.6	M	0.99	1.17
Goodwin and Meeuwsen (30)	*N* = 30	26.2 SD = 8.0	F	0.47	0.34
Porter and Magill (31)	*N* = 60	N. R.	M = 18 F = 42	0.57	0.32
Schmidt et al. (32)	*N* = 42	24.1 SD = 3.6	M = 30 F = 12	0.48	0.37
Studies examining performance changes in golf-specific motor skills as result of different forms of augmented feedback					
An et al. (33)	*N* = 30	22.4 SD = 1.41	M = 20 F = 10	0.59	0.36
Biénkiewicz et al. (82)	*N* = 30	19.6 SD = 2.4	M = 24, F = 6	0.96	0.68
Butki and Hoffman (34)	*N* = 78	N. R.	M = 48 F = 30	0.99	1.03
Chiviacowsky et al. (35)	*N* = 28	23.2 SD = 6.71	M = 14 F = 14	0.66	0.47
de Souza Nunes et al. (36)	*N* = 40	69 SD = 2.95	M = 18 F = 22	1	1.03
Guadagnoli et al. (37)	*N* = 30	29–50	N. R.	0.89	0.56
Jalalvand et al. (38)	*N* = 60	20.92 SD = 1.59	M = 32 F = 28	0.93	0.34
Post et al. (40)	*N* = 44	21.8 SD = 1.3	M = 6 F = 38	0.86	0.47
Pourbehbahani et al. (39)	*N* = 40	26.10 SD = 5.56	M = 20 F = 20	0.96	0.53
Ring et al. (41)	*N* = 24	22	M	0.93	0.64
Smith et al. (42)	*N* = 48	22.1 SD = 3.6	M = 24 F = 24	0.99	0.60
Studies examining performance changes in golf-specific motor skills as result of implicit and explicit learning					
Chauvel et al. (43)	*N* = 96	23.5 SD = 3.3, 65 SD = 3.7	M = 49 F = 47	0.61	0.35
Hardy et al. (44)	*N* = 32	21.23 SD = N. R.	M = 16 F = 16	0.58	0.43
Lam et al. (45)	*N* = 36	21.49 SD = 2.03	M = 22 F = 14	0.99	0.68
Masters (46)	*N* = 40	27.22	N. R.	0.75	0.43
Maxwell et al. (47)	*N* = 27	22.81 SD = 2.17	N. R.	0.22	0.24
Maxwell et al. (48)	*N* = 29	20.86 SD = 2.4	N. R.	0.88	0.68
Moore et al. (49)	*N* = 40	19.55 SD = 1.65	N. R.	0.58	0.28
Poolton et al. (50)	*N* = 35	21.1 SD = 1.48	M = 11 F = 24	0.36	0.72
Vine et al. (51)	*N* = 22	20.95 SD = 2.66	M	0.25	0.23
Vine et al. (52)	*N* = 45	21.22 SD = 4.41	N. R.	0.77	0.38
Zhu et al. (53)	*N* = 18	22	N. R.	0.62	0.49
Studies examining performance changes in golf-specific motor skills as result of different foci of attention					
Aiken and Becker (54)	*N* = 79	19.28 SD = 2.31	M = 27 F = 52	0.58	0.28
An et al. (55)	*N* = 24	27.3 SD = 2.05	M	0.29	0.58
Bell and Hardy (56)	*N* = 33	37.06 SD = 17.84	M	0.99	0.73
Brocken et al. (57)	*N* = 60	8.94 SD = 0.45, 11.66 SD = 0.43	M = 26 F = 34	0.88	0.36
Christina and Alpenfels (58)	*N* = 45	65 SD = 7.79	M	0.53	0.52
Christina and Alpenfels (58)	*N* = 39	64 SD = 15	M	0.23	0.38
Land et al. (59)	*N* = 30	47.93 SD = 14.37	M	0.99	0.75
Lawrence et al. (60)	*N* = 29	22.1 SD = 4.3	N. R.	0.48	0.35
Munzert et al. (61)	*N* = 30	N. R.	M = 9 F = 21	0.90	0.54
Perkins-Ceccato et al. (62)	*N* = 20	N. R.	M = 16 F = 4	0.79	0.57
Poolton et al. (63)	*N* = 30	24.1 SD = 5.94	M = 7 F = 23	0.31	0.39
Wulf et al. (64)	*N* = 22	21–29	M = 13 F = 9	0.97	0.72
Wulf and Su (65)	*N* = 30	N. R.	N. R.	0.65	0.44
Wulf and Su (65)	*N* = 6	N. R.	N. R.	0.30	0.98

N. R., not reported.

Three studies investigated the effects of QE as a form of implicit learning (49, 51, 52). All studies reported significant advantages compared to either a control group, an explicit learning group, or even another implicit learning group (analogy learning). Although the results of these studies support QE as an implicit learning method, there is still no clear consensus on the uniqueness of QE as a learning method on its own. Lee and Schmidt have discussed this extensively in their review (7) and suggest that QE may lead to improved performance due to a clear external focus associated with the instruction. Further research is necessary to determine whether QE should be considered a learning method that favors implicit learning processes or a specific instruction that effectively creates an external focus.

As mentioned above, further studies are needed to provide a clearer picture on the effects of implicit vs. explicit learning. Even though most motor learning literature considers implicit learning to be most beneficial for motor skill learning, this learning strategy is still very much debated [for review see (112)]. Proponents and pioneers of implicit motor learning and the research behind it disagree on whether the benefits are genuine or are related to the abilities of the practitioner. In this context, two studies particularly advocate the use of explicit techniques with implicit features as they are more practical and applicable (50, 113). With regard to implicit vs. explicit motor learning in golf, three studies compared both methods and their effect on putting (44, 46, 47), with either no difference to a control group or no difference at all between the two learning methods, although effect sizes were considerable (Table 7). Therefore, it seems possible that the lack of statistical significance may result from low power which does not necessarily indicate that the learning methods are not effective for golf. This is of importance, as according to the specific demands of the play on the course, an implicit learning method should be favorable to perform better during dual-task situations or under pressure [for review see (114)]. It should be noted that although Cabral et al. (114) expect better performance from implicit learning than from explicit learning (especially under pressure). However, the studies in their review were at large of low statistical power, and the evidence should therefore be interpreted carefully. Furthermore, in a recent comment Bobrownicki et al. (14) express concerns about the systematic review by Cabral et al. (114), citing a lack of clarity of objectives and methodology and unrepresentative comparison groups (14). In conclusion, there is no clear picture on advantages and disadvantages of implicit vs. explicit in golf and it would be interesting to see more studies with greater numbers of participants that investigate this learning strategy with golf-specific motor skills.

### 4.5 Cognitive training

One other learning strategy with an inconclusive outcome is “cognitive training”. Cognitive training includes a plethora of different methods, from visual occlusion to perceptual-cognitive training and mental imaging. Such variety makes this learning strategy a broad field to be investigated in detail. In golf we found 12 studies comparing specific cognitive learning methods to increase putting performance. Five studies examined the effects of visual illusion and demonstrated improved performance when perceiving a larger hole during retention (15, 17, 22) or transfer (24, 25). Another study looking at occluded vision and imagery (21) found increased performance for all intervention groups, but no significant differences between them. Four studies investigated motor imagery (both positive and negative) and action observation but found inconclusive results (16, 18, 19, 23). Since one of the studies showed a more significant deterioration in performance with negative imaging for novices compared to high level players (23), we again recommend looking at the effects of the different strategies at different skill levels before deriving any specific learning recommendations. However, in line with the basic literature on the benefits of cognitive learning in general, we can recommend enriching the learning process with one or more methods from cognitive training. This is especially supported by the fact that different reviews in the area of neurorehabilitation have already shown a positive effect of cognitive or mental training (115, 116). In a more specific review on motor-skill learning, Schuster et al. (117) showed that mental imagery interventions involving individual, supervised sessions after physical practice are beneficial for both genders, especially in younger adults [for review see (117)]. Therefore, future studies should aim to clarify the possible superiority of some methods over others and look at the effect of promising combinations of different training methods.

### 4.6 Limitations

The fact that we found inconclusive results in some of the learning strategies could be attributed to the less-than-optimal classification of studies into the cognitive learning strategy that we outlined in our review. In fact, it was not always possible to categorize certain studies into the learning strategies we selected according to Schmidt et al. (1). To allow a grouping of studies that do not fit the motor learning strategies that we chose, we recommend turning to the aforementioned OPTIMAL theory of learning by Wulf and Lewthwaite (10). Recently, it has been suggested that better learning success might be expected as a result of a combination of several strategies and methods (118). To our knowledge, An et al. (33) are the first to try a combination of motor learning strategies in order to enhance golf putting performance (119). They suggest that the motivational and attentional factors of the OPTIMAL theory of learning can be applied both separately and in combination during practice to enhance motor skill learning.

The OPTIMAL theory of learning states that learning is facilitated by: (1) conditions that enhance expectancies for future performance; (2) variables that influence one's autonomy; and (3) an external focus of attention. The first can be achieved through enhanced self-efficacy, increased task interest, higher satisfaction with one's abilities and performance, as well as greater positive affect. A number of studies in the present review incorporate these aspects, and further support the ideas of Wulf and Lewthwaite. Those studies that showed the impact of the perception of a larger hole on the number of successful putts reveal a decrease in the perceived task difficulty. This reduction forms higher expectations of success and increases the number of successful putts (15, 17, 22, 24, 25). Similarly, studies from other strategies must be considered when it comes to enhanced expectancies, as for example positive feedback (35) leads to higher self-efficacy as well and the reduction of errors also affects the expected movement outcome (45, 48). The second aspect of the OPTIMAL theory of learning proposes that variables fostering a learner's autonomy are beneficial, as they provide one with control or a choice over the task at hand. Seven studies in our review provided their participants with a “choice” opportunity like self-chosen feedback, the possibility to choose the color of their balls or placing a visual aid around the hole, with some showing promising results (20, 26, 33, 36–40). We have extensively discussed studies investigating an external focus in golf, other sports, or various other research areas. However, connections can also be made from other motor learning strategies to this third aspect of the OPTIMAL theory. For example, Beilock and colleagues (16) and Taylor and Shaw (23) look at positive images that focus on the outcome of the movement. This further shows that a classification of one study into only one strategy is not necessarily possible and that different aspects of different strategies and theoretical models can also be analyzed in one study.

A publication pre-dating the OPTIMAL Theory is “The Challenge Point Framework” (12). The Framework can be possibly used as a guiding tool for effective golf training. It takes into account factors such as task difficulty, and the organization of training to optimize learning. Various publications have incorporated these suggestions or mentioned the Framework in their studies on motor learning, motor control, rehabilitation or cognitive development (31, 81, 120–127). We can use the Framework to explain some limitations and problems that arise in golf-related motor learning research to date. For example, a player's performance on the practice range does not necessarily predict how well he will play on the course and a participant's performance during acquisition does not necessarily transfer to a retention or follow-up task. Therefore, one aim for future studies must be to design practice protocols that optimize the relationship between practice and transfer. Future research should also aim at manipulating the level of difficulty during skill learning to investigate how challenge contributes to effective motor learning in golf-related tasks. The combination of the OPTIMAL Theory of learning and the Challenge Point Framework along with the results of the individual motor learning strategies we have discussed can shed new light on golf-related motor learning research and will help us make actual recommendations for players, coaches and researchers in the near future.

The biggest limitation of the present review, in our opinion, is the lack of statistical power for more than half (29 out of 52) of the studies included in the present review. The results of our *post-hoc* power analysis (Table 6) show that, except for the studies looking at forms of augmented feedback, no other motor learning strategy has a mean statistical power over .8. However, even though there is an overall lack of power for the different motor learning strategy groups, at the single study level we see a total of 23 studies reaching the power-threshold of >.8 (Table 7). Following the review of Dumas-Mallet et al. (128), a lack of power on a single paper level, as well as in reviews, unfortunately seems to be quite common in various fields of research. Nevertheless, the presentation and interpretation of results must be considered very carefully, as low statistical power (e.g., due to small sample size of studies, small effects, or both) makes it unlikely to detect the real effect that might be present. Thus, we strongly advise researchers to adjust the number of participants included in their studies to reach at least a power of .8 (potential Type II error of 20%). As a practical tip, we suggest reducing the number of groups (without sacrificing the control group) if only a certain number of participants can be included in the study.

This systematic review included a rather small number of RCTs for certain subcategories, with an overall large heterogeneity in participant characteristics across studies. In addition, there is a large variation in the extent of practice between studies, which further increases the heterogeneity between studies. Furthermore, except for very few exceptions, the studies investigated the effects of motor learning on putting (43 out of 52) in novice players (41 out of 52). Thus, it must be acknowledged that the results might be of limited relevance for semi-professional or professional golf athletes and also for amateur golfers regardless of their level of performance (129). Previous research mostly refers to distance and accuracy as of the most important parameters to represent performance in golf (130). However, in a real game, distance is not always synonymous with the desired result of a shot, as various factors can influence the (required) distance of a shot on a course. Therefore, further research should continue include other co-factors to quantify actual performance beside distance. To date, most motor learning studies are either laboratory-based or a simplified version of their “real” task. Sigrist et al. (81) discuss this issue in their review and point out the lack of transferability from research results towards real life. They therefore propose using simulators together with augmented feedback to provide realistic environments if a real-life setup is not feasible (e.g., due to technical or costly reasons). We strongly believe that using such simulators will help transfer the motor skills acquired in the laboratory to the real environment and more complex situations, thus creating a lab-to-life approach. There is a clear need for motor learning studies investigating not only the differences in skill level but also the effects of different motor learning strategies on the full swing, with both irons and woods. Most of the studies were clearly designed to study motor learning through golf rather than for golf [see (131) and (14)], limiting their ecological validity for the applied field. Finally, the selection of studies itself represents a limiting factor for our systematic review. The problems associated with the variety of motor learning strategies and the poor comparability of control variables make a classical approach according to the PICO framework and the PRISMA guidelines difficult. However, we believe that by addressing the fundamental problems of the studies, we can lay a foundation for further research that will allow for comparability and generalizability of results in the future, and thus be helpful to players, coaches, and researchers alike.

### 4.7 Conclusion

Notwithstanding the fact that our results of motor learning strategies in golf underpin and extend the general ideas on how to structure the learning process of motor skills, it is currently not possible to derive specific training and learning recommendations for golfers and golf coaches. Due to the limitations of the current research, we can only presume recommendations on how to structure the learning process for “attentional focus”, “augmented feedback”, “practice scheduling” and “implicit and explicit learning”. Superior methods within these learning strategies seem to be an external focus of attention, increasing contextual interference, distributed practice, as well as exercises that aim to reduce the number of errors during the initial practice of golf-specific motor skills.

The present review supports the ideas behind the Challenge Point Framework (optimal interaction between the skill-level of the learner and functional difficulty of the learning task) as well as the OPTIMAL theory of learning (advantage of an external focus of attention, enhanced expectancy for future performance and a high degree of autonomy during the learning process) for learning golf-specific motor skills. From a motor control point of view, the premises underlying the OPTIMAL theory of learning might be well aligned with a higher degree of automation during the execution of a motor skill. However, further research is needed to generalize this assumption to other sports and motor tasks and to understand the potential moderating factors that may influence the effectiveness of the mechanisms that underly this theory. Moreover, there is a need to investigate further the potential interactions between different learning strategies and methods and how they should be composed to facilitate optimal learning. In addition, the influence of skill-level, as well as the required level of “challenge” should be considered in more detail.

In order to cope with the complexity of motor learning in golf, we propose, as a next step, to investigate different combinations of these motor learning strategies and their methods, especially those for which we were able to already aggregate preliminary evidence, in players of different skill levels. Additionally, we suggest conducting RCTs over a longer period of time, as it cannot be taken for granted that the short-term effects that are classically observed after one or two acquisition phases necessarily translate into similar benefits in the long term.

## Data Availability

The original contributions presented in the study are included in the article/**Supplementary Material**, further inquiries can be directed to the corresponding author.

## References

[1] Schmidt R Lee T Winstein C Wulf G Zelaznik H. Motor control and learning: a behavioral emphasis. In Human Kinetics, 6th Edn. Champaign, IL: Human Kinetics (2018).

[2] Sinclair J Currigan G Fewtrell DJ Taylor PJ. Biomechanical correlates of club-head velocity during the golf swing. Int J Perform Anal Sport. (2017) 14(1):54–63. 10.1080/24748668.2014.11868702

[3] Sprigings EJ Neal RJ. An insight into the importance of wrist torque in driving the golfball: a simulation study. J Appl Biomech. (2000) 16(4):356–66. 10.1123/JAB.16.4.356

[4] Guadagnoli MA Bertram CP. Optimizing practice for performance under pressure. Int J Golf Sci. (2014) 3(2):119–27. 10.1123/ijgs.2014-0021

[5] Guadagnoli MA Lindquist K. Challenge point framework and efficient learning of golf. Int J Sports Sci Coach. (2007) 2(1_suppl):185–97. 10.1260/174795407789705505

[6] Keogh JWL Hume PA. Evidence for biomechanics and motor learning research improving golf performance. Sports Biomech. (2012) 11(2):288–309. 10.1080/14763141.2012.671354 22900408

[7] Lee T Schmidt R. Par (plan-act-review) golf: motor learning research and improving golf skills. Int J Golf Sci. (2014) 3(1):2–25. 10.1123/ijgs.2014-0004

[8] Moffat D Collins D Carson HJ. Target versus ball focused aiming when putting: what has been done and what has been missed. Int J Golf Sci. (2017) 6(1):35–55. 10.1123/IJGS.2017-0002

[9] Wulf G Orr S Chauvel G. Optimizing golf skill learning. In: Toms M, editor. Routledge International Handbook of Golf Science. London: Routledge (2017). pp. 88–97. 10.4324/9781315641782-10

[10] Wulf G Lewthwaite R. Optimizing performance through intrinsic motivation and attention for learning: the OPTIMAL theory of motor learning. Psychon Bull Rev. (2016) 23(5):1382–414. 10.3758/S13423-015-0999-9 26833314

[11] Magill R Anderson D. Motor Learning and Control: Concepts and Applications, 12th Edn. New York: McGraw Hill LLC (2021).

[12] Guadagnoli MA Lee TD. Challenge point: a framework for conceptualizing the effects of various practice conditions in motor learning. J Mot Behav. (2004) 36(2):212–24. 10.3200/JMBR.36.2.212-224 15130871

[13] Page MJ McKenzie JE Bossuyt PM Boutron I Hoffmann TC Mulrow CD The PRISMA 2020 statement: an updated guideline for reporting systematic reviews. Br Med J. (2021) 372:n71. 10.1136/BMJ.N71 33782057 PMC8005924

[14] Bobrownicki R Carson HJ Collins D. Conducting systematic reviews of applied interventions: a comment on Cabral et al. (2022). Sport Exerc Perform Psychol. (2022) 11(3), 264–74. 10.1037/SPY0000299

[15] Bahmani M Wulf G Ghadiri F Karimi S Lewthwaite R. Enhancing performance expectancies through visual illusions facilitates motor learning in children. Hum Mov Sci. (2017) 55:1–7. 10.1016/j.humov.2017.07.001 28709045

[16] Beilock SL Afremow JA Rabe AL Carr TH. “Don’t miss!” the debilitating effects of suppressive imagery on golf putting performance. J Sport Exerc Psychol. (2001) 23(3):200–21. 10.1123/jsep.23.3.200

[17] Chauvel G Wulf G Maquestiaux F. Visual illusions can facilitate sport skill learning. Psychon Bull Rev. (2015) 22(3):717–21. 10.3758/s13423-014-0744-9 25316049

[18] Frank C Land WM Popp C Schack T. Mental representation and mental practice: experimental investigation on the functional links between motor memory and motor imagery. PLoS One. (2014) 9(4):e95175. 10.1371/journal.pone.0095175 24743576 PMC3990621

[19] Kim T Frank C Schack T. A systematic investigation of the effect of action observation training and motor imagery training on the development of mental representation structure and skill performance. Front Hum Neurosci. (2017) 11:499. 10.3389/fnhum.2017.00499 29089881 PMC5650990

[20] Lewthwaite R Chiviacowsky S Drews R Wulf G. Choose to move: the motivational impact of autonomy support on motor learning. Psychon Bull Rev. (2015) 22(5):1383–8. 10.3758/s13423-015-0814-7 25732095

[21] Meacci WG Pastore DL. Effects of occluded vision and imagery on putting golf balls. Percept Mot Skills. (1995) 80(1):179–86. 10.2466/pms.1995.80.1.179 7624190

[22] Palmer K Chiviacowsky S Wulf G. Enhanced expectancies facilitate golf putting. Psychol Sport Exerc. (2016) 22:229–32. 10.1016/J.PSYCHSPORT.2015.08.009

[23] Taylor JA Shaw DF. The effects of outcome imagery on golf-putting performance. J Sports Sci. (2002) 20(8):607–13. 10.1080/026404102320183167 12190280

[24] Ziv G Ochayon M Lidor R. Enhanced or diminished expectancies in golf putting—which actually affects performance? Psychol Sport Exerc. (2018) 40:82–6. 10.1016/J.PSYCHSPORT.2018.10.003

[25] Ziv G Lidor R Lavie M. Enhanced expectancies in golf putting—a replication study with increased ecological validity. Int J Sport Exerc Psychol. (2019) 19(1):1–12. 10.1080/1612197X.2019.1637362

[26] Ziv G Lidor R Elbaz L Lavie M. Preference-performance dissociation in golf putting. Front Psychol. (2020) 11:102. 10.3389/fpsyg.2020.00102 32116914 PMC7025568

[27] Chua LK Dimapilis MK Iwatsuki T Abdollahipour R Lewthwaite R Wulf G. Practice variability promotes an external focus of attention and enhances motor skill learning. Hum Mov Sci. (2019) 64:307–19. 10.1016/j.humov.2019.02.015 30831389

[28] Dail TK Christina RW. Distribution of practice and metacognition in learning and long-term retention of a discrete motor task. Res Q Exerc Sport. (2004) 75(2):148–55. 10.1080/02701367.2004.10609146 15209333

[29] Fazeli D Taheri H Saberi Kakhki A. Random versus blocked practice to enhance mental representation in golf putting. Percept Mot Skills. (2017) 124(3):674–88. 10.1177/0031512517704106 28449601

[30] Goodwin JE Meeuwsen HJ. Investigation of the contextual interference effect in the manipulation of the motor parameter of over-all force. Percept Mot Skills. (1996) 85(3 PART 1):735–43. 10.2466/pms.1996.83.3.735 8961310

[31] Porter JM Magill RA. Systematically increasing contextual interference is beneficial for learning sport skills. J Sports Sci. (2010) 28(12):1277–85. 10.1080/02640414.2010.502946 20845219

[32] Schmidt M Kemena M Jaitner T. Null effects of different amounts of task variation in both contextual interference and differential learning paradigms. Percept Mot Skills. (2021) 128(4):1836–50. 10.1177/00315125211022302 34078209

[33] An J Lewthwaite R Lee S Wulf G. Choice of practice-task order enhances golf skill learning. Psychol Sport Exerc. (2020b) 50:101737. 10.1016/j.psychsport.2020.101737

[34] Butki BD Hoffman SJ. Effects of reducing frequency of intrinsic knowledge of results on the learning of a motor skill. Percept Mot Skills. (2003) 97(2):569–80. 10.2466/pms.2003.97.2.569 14620246

[35] Chiviacowsky S Harter NM Gonçalves GS Cardozo PL. Temporal-comparative feedback facilitates golf putting. Front Psychol. (2018) 9(JAN):2691. 10.3389/fpsyg.2018.02691 30662424 PMC6328491

[36] de Souza Nunes ME Correa UC De Souza MGTX Basso L Coelho DB Santos S. No improvement on the learning of golf putting by older persons with self-controlled knowledge of performance. J Aging Phys Act. (2019) 27(3):300–8. 10.1123/japa.2018-0053 30160582

[37] Guadagnoli MA Holcomb W Davis M. The efficacy of video feedback for learning the golf swing. J Sports Sci. (2002) 20(8):615–22. 10.1080/026404102320183176 12190281

[38] Jalalvand M Bahram A Daneshfar A Arsham S. The effect of gradual self-control of task difficulty and feedback on learning golf putting. Res Q Exerc Sport. (2019) 90(4):429–39. 10.1080/02701367.2019.1612510 31329023

[39] Pourbehbahani Z Saemi E Cheng M-Y Dehghan MR. Both sensorimotor rhythm neurofeedback and self-controlled practice enhance motor learning and performance in novice golfers. Behav Sci (Basel, Switzerland). (2023) 13(1):65. 10.3390/bs13010065 PMC9854920 36661637

[40] Post PG Aiken CA Laughlin DD Fairbrother JT. Self-control over combined video feedback and modeling facilitates motor learning. Hum Mov Sci. (2016) 47:49–59. 10.1016/j.humov.2016.01.014 26874750

[41] Ring C Cooke A Kavussanu M McIntyre D Masters R. Investigating the efficacy of neurofeedback training for expediting expertise and excellence in sport. Psychol Sport Exerc. (2015) 16(P1):118–27. 10.1016/J.PSYCHSPORT.2014.08.005

[42] Smith PJK Taylor SJ Withers K. Applying bandwidth feedback scheduling to a golf shot. Res Q Exerc Sport. (1997) 68(3):215–21. 10.1080/02701367.1997.10608000 9294875

[43] Chauvel G Maquestiaux F Hartley AA Joubert S Didierjean A Masters RSW. Age effects shrink when motor learning is predominantly supported by nondeclarative, automatic memory processes: evidence from golf putting. Q J Exp Psychol. (2012) 65(1):25–38. 10.1080/17470218.2011.588714 21736434

[44] Hardy L Mullen R Jones G. Knowledge and conscious control of motor actions under stress. Br J Psychol. (1996) 87(4):621–36. 10.1111/j.2044-8295.1996.tb02612.x 8962480

[45] Lam WK Maxwell JP Masters RSW. Probing the allocation of attention in implicit (motor) learning. J Sports Sci. (2010) 28(14):1543–54. 10.1080/02640414.2010.517543 21049315

[46] Masters RSW. Knowledge, knerves and know-how: the role of explicit versus implicit knowledge in the breakdown of a complex motor skill under pressure. Br J Psychol. (1992) 83(3):343–58. 10.1111/j.2044-8295.1992.tb02446.x

[47] Maxwell JP Masters RSW Eves FF. From novice to no know-how: a longitudinal study of implicit motor learning. J Sports Sci. (2000) 18(2):111–20. 10.1080/026404100365180 10718567

[48] Maxwell JP Masters RSW Kerr E Weedon E. The implicit benefit of learning without errors. Q J Exp Psychol A. (2001) 54(4):1049–68. 10.1080/713756014 11765732

[49] Moore LJ Vine SJ Cooke A Ring C Wilson MR. Quiet eye training expedites motor learning and aids performance under heightened anxiety: the roles of response programming and external attention. Psychophysiology. (2012) 49(7):1005–15. 10.1111/j.1469-8986.2012.01379.x 22564009

[50] Poolton JM Masters RSW Maxwell JP. The relationship between initial errorless learning conditions and subsequent performance. Hum Mov Sci. (2005) 24(3):362–78. 10.1016/j.humov.2005.06.006 16087262

[51] Vine SJ Moore LJ Wilson MR. Quiet eye training facilitates competitive putting performance in elite golfers. Front Psychol. (2011) 2(JAN):8. 10.3389/FPSYG.2011.00008/BIBTEX 21713182 PMC3111367

[52] Vine SJ Moore LJ Cooke A Ring C Wilson MR. Quiet eye training: a means to implicit motor lear. Int J Sport Psychol. (2013) 44(4):367–86. 10.7352/IJSP.2013.44.367

[53] Zhu FF Poolton JM Wilson MR Maxwell JP Masters RSW. Neural co-activation as a yardstick of implicit motor learning and the propensity for conscious control of movement. Biol Psychol. (2011) 87(1):66–73. 10.1016/j.biopsycho.2011.02.004 21315795

[54] Aiken CA Becker KA. Utilising an internal focus of attention during preparation and an external focus during execution may facilitate motor learning. Eur J Sport Sci. (2023) 23(2):259–66. 10.1080/17461391.2022.2042604 35164654

[55] An J Wulf G Kim S. Increased carry distance and x-factor stretch in golf through an external focus of attention. J Mot Learn Dev. (2013) 1(1):2–11. 10.1123/jmld.1.1.2

[56] Bell JJ Hardy J. Effects of attentional focus on skilled performance in golf. J Appl Sport Psychol. (2009) 21(2):163–77. 10.1080/10413200902795323

[57] Brocken JEA Kal EC van der Kamp J. Focus of attention in children’s motor learning: examining the role of age and working memory. J Mot Behav. (2016) 48(6):527–34. 10.1080/00222895.2016.1152224 27340947

[58] Christina B Alpenfels E. Influence of attentional focus on learning a swing path change. Int J Golf Sci. (2014) 3(1):35–49. 10.1123/ijgs.2014-0001

[59] Land WM Tenenbaum G Ward P Marquardt C. Examination of visual information as a mediator of external focus benefits. J Sport Exerc Psychol. (2013) 35(3):250–9. 10.1123/jsep.35.3.250 23798588

[60] Lawrence GP Gottwald VM Khan MA Kramer RS. The movement kinematics and learning strategies associated with adopting different foci of attention during both acquisition and anxious performance. Front Psychol. (2012) 3(NOV):468. 10.3389/fpsyg.2012.00468 23130008 PMC3487420

[61] Munzert J Maurer H Reiser M. Verbal-motor attention-focusing instructions influence kinematics and performance on a golf-putting task. J Mot Behav. (2014) 46(5):309–18. 10.1080/00222895.2014.912197 24857254

[62] Perkins-Ceccato N Passmore SR Lee TD. Effects of focus of attention depend on golfers’ skill. J Sports Sci. (2003) 21(8):593–600. 10.1080/0264041031000101980 12875310

[63] Poolton JM Maxwell JP Masters RSW Raab M. Benefits of an external focus of attention: common coding or conscious processing? J Sports Sci. (2006) 24(1):89–99. 10.1080/02640410500130854 16368617

[64] Wulf G Lauterbach B Toole T. The learning advantages of an external focus of attention in golf. Res Q Exerc Sport. (1999) 70(2):120–6. 10.1080/02701367.1999.10608029 10380243

[65] Wulf G Su J. An external focus of attention enhances golf shot accuracy in beginners and experts. Res Q Exerc Sport. (2007) 78(4):384–9. 10.1080/02701367.2007.10599436 17941543

[66] Higgins JPT Thomas J Chandler J Cumpston M Li T Page MJ Cochrane Handbook for Systematic Reviews of Interventions. Chichester: The Cochrane Collaboration and John Wiley & Sons Ltd. (2019). p. 1–694. 10.1002/9781119536604

[67] Cohen J. Statistical power analysis for the behavioral sciences. In: Statistical Power Analysis for the Behavioral Sciences. New York: Routledge (2013). p. 1–17. 10.4324/9780203771587

[68] Driskell JE Copper C Moran A. Does mental practice enhance performance? J Appl Psychol. (1994) 79(4):481–92. 10.1037/0021-9010.79.4.481

[69] Gavelin HM Dong C Minkov R Bahar-Fuchs A Ellis KA Lautenschlager NT Combined physical and cognitive training for older adults with and without cognitive impairment: a systematic review and network meta-analysis of randomized controlled trials. Ageing Res Rev. (2021) 66:101232. 10.1016/J.ARR.2020.101232 33249177

[70] Bherer L. Cognitive plasticity in older adults: effects of cognitive training and physical exercise. Ann N Y Acad Sci. (2015) 1337(1):1–6. 10.1111/nyas.12682 25773610

[71] Savikangas T Törmäkangas T Tirkkonen A Alen M Fielding RA Kivipelto M The effects of a physical and cognitive training intervention vs. physical training alone on older adults’ physical activity: a randomized controlled trial with extended follow-up during COVID-19. PLoS One. (2021) 16(10 October):2–17. 10.1371/journal.pone.0258559 PMC8513828 34644357

[72] Walton CC Keegan RJ Martin M Hallock H. The potential role for cognitive training in sport: more research needed. Front Psychol. (2018) 9(JUL):1121. 10.3389/fpsyg.2018.01121 30018585 PMC6037849

[73] Di Corrado D Guarnera M Guerrera CS Maldonato NM Di Nuovo S Castellano S Mental imagery skills in competitive young athletes and non-athletes. Front Psychol. (2020) 11:633. 10.3389/FPSYG.2020.00633 32362857 PMC7180224

[74] Simonsmeier BA Andronie M Buecker S Frank C. The effects of imagery interventions in sports: a meta-analysis. Int Rev Sport Exerc Psychol. (2021) 14(1):186–207. 10.1080/1750984X.2020.1780627

[75] Ste-Marie DM Law B Rymal AM Jenny O Hall C McCullagh P. Observation interventions for motor skill learning and performance: an applied model for the use of observation. Int Rev Sport Exerc Psychol. (2012) 5(2):145–76. 10.1080/1750984X.2012.665076

[76] Woodman T Hardy L. The relative impact of cognitive anxiety and self-confidence upon sport performance: a meta-analysis. J Sports Sci. (2003) 21(6):443–57. 10.1080/0264041031000101809 12846532

[77] Witt JK Linkenauger SA Proffitt DR. Get me out of this slump! visual illusions improve sports performance. Psychol Sci. (2012) 23(4):397–9. 10.1177/0956797611428810 22395130

[78] Kwon YH Kwon JW Lee MH. Effectiveness of motor sequential learning according to practice schedules in healthy adults; istributed practice versus massed practice. J Phys Ther Sci. (2015) 27(3):769–72. 10.1589/jpts.27.769 25931727 PMC4395711

[79] Shea JB Morgan RL. Contextual interference effects on the acquisition, retention, and transfer of a motor skill. J Exp Psychol Hum Learn Mem. (1979) 5(2):179–87. 10.1037/0278-7393.5.2.179

[80] Schmidt R Lee T. Motor Learning and Performance: from Principles to Application, 6th Edn. Champaign: Human Kinetics (2020).

[81] Sigrist R Rauter G Riener R Wolf P. Augmented visual, auditory, haptic, and multimodal feedback in motor learning: a review. Psychon Bull Rev. (2013) 20(1):21–53. 10.3758/s13423-012-0333-8 23132605

[82] Bieńkiewicz M Bringoux L Buloup F Rodger M Craig C Bourdin C. The limitations of being a copycat: learning golf putting through auditory and visual guidance. Front Psychol. (2019) 10(FEB):92. 10.3389/fpsyg.2019.00092 30800082 PMC6376899

[83] Kümmel J Kramer A Gruber M. Robotic guidance induces long-lasting changes in the movement pattern of a novel sport-specific motor task. Hum Mov Sci. (2014) 38:23–33. 10.1016/j.humov.2014.08.003 25238621

[84] Chiviacowsky S Drews R. Temporal-comparative feedback affects motor learning. J Mot Learn Dev. (2016) 4(2):208–18. 10.1123/JMLD.2015-0034

[85] Chiviacowsky S Wulf G. Feedback after good trials enhances learning. Res Q Exerc Sport. (2013) 78(2):40–7. 10.1080/02701367.2007.10599402 17479573

[86] Chiviacowsky S Wulf G. Self-controlled feedback: does it enhance learning because performers get feedback when they need it? Res Q Exerc Sport. (2013) 73(4):408–15. 10.1080/02701367.2002.10609040 12495242

[87] Capio CM Poolton JM Sit CHP Eguia KF Masters RSW. Reduction of errors during practice facilitates fundamental movement skill learning in children with intellectual disabilities. J Intellect Disabil Res. (2013) 57(4):295–305. 10.1111/J.1365-2788.2012.01535.X 22369034

[88] Chua LK Jimenez-Diaz J Lewthwaite R Kim T Wulf G. Superiority of external attentional focus for motor performance and learning: systematic reviews and meta-analyses. Psychol Bull. (2021) 147(6):618–45. 10.1037/BUL0000335 34843301

[89] Wulf G. Attentional focus and motor learning: a review of 15 years. Int Rev Sport Exerc Psychol. (2013) 6(1):77–104. 10.1080/1750984X.2012.723728

[90] Lohse KR Sherwood DE Healy AF. How changing the focus of attention affects performance, kinematics, and electromyography in dart throwing. Hum Mov Sci. (2010) 29(4):542–55. 10.1016/J.HUMOV.2010.05.001 20541275

[91] Al-Abood SA Bennett SJ Hernandez FM Ashford D Davids K. Effect of verbal instructions and image size on visual search strategies in basketball free throw shooting. J Sport Sci. (2010) 20(3):271–8. 10.1080/026404102317284817 11999481

[92] Zachry T Wulf G Mercer J Bezodis N. Increased movement accuracy and reduced EMG activity as the result of adopting an external focus of attention. Brain Res Bull. (2005) 67(4):304–9. 10.1016/j.brainresbull.2005.06.035 16182938

[93] Abdollahipour R Wulf G Psotta R Palomo Nieto M. Performance of gymnastics skill benefits from an external focus of attention. J Sports Sci. (2015) 33(17):1807–13. 10.1080/02640414.2015.1012102 25774536

[94] Hunt C Paez A Folmar E. The impact of attentional focus on the treatment of muscoloskeletal and movement disorders. Int J Sports Phys Ther. (2017) 12(6):901. 10.26603/ijspt20170901 29158952 PMC5675366

[95] Song JH. The role of attention in motor control and learning. Curr Opin Psychol. (2019) 29:261–5. 10.1016/J.COPSYC.2019.08.002 31491612

[96] McKay B Corson AE Seedu J De Faveri CS Hasan H Arnold K Reporting bias, not external focus. SportRxiv. (2023). 10.51224/SRXIV.304 39480294

[97] Herrebrøden H. Motor performers need task-relevant information: proposing an alternative mechanism for the attentional focus effect. J Mot Behav. (2023) 55(1):125–34. 10.1080/00222895.2022.2122920 36104021

[98] Mödinger M Woll A Wagner I. Video-based visual feedback to enhance motor learning in physical education—a systematic review. Ger J Exerc Sport Res. (2021) 52(1):1–14. 10.1007/s12662-021-00782-y

[99] Lee YF Altschuld JW Chiang FS Yue CSJ Sung HT Chang CH. Effects of augmented feedback with error self-estimates on vocational high school Students’ motor skill learning. Vocat Learn. (2022) 15(1):1–20. 10.1007/S12186-021-09273-5/TABLES/4

[100] Storberget M Grødahl LHJ Snodgrass S Vliet P Van Heneghan N. Verbal augmented feedback in the rehabilitation of lower extremity musculoskeletal dysfunctions: a systematic review. BMJ Open Sport Exerc Med. (2017) 3(1):e000256. 10.1136/BMJSEM-2017-000256 29018544 PMC5623330

[101] Guadagnoli MA Dornier LA Tandy RD. Optimal length for summary knowledge of results: the influence of task-related experience and complexity. Res Q Exerc Sport. (1996) 67(2):239–48. 10.1080/02701367.1996.10607950 8836005

[102] Moinuddin A Goel A Sethi Y. The role of augmented feedback on motor learning: a systematic review. Cureus. (2021) 13(11):1–4. 10.7759/CUREUS.19695 PMC8681883 34976475

[103] Feghhi I Valizade R. Systematically increasing contextual interference is beneficial for learning single task. Procedia Soc Behav Sci. (2011) 30:2191–3. 10.1016/J.SBSPRO.2011.10.425

[104] Levin MF Demers M. Motor learning in neurological rehabilitation. Disabil Rehabil. (2021) 43(24):3445–53. 10.1080/09638288.2020.1752317 32320305

[105] Giboin LS Tokuno C Kramer A Henry M Gruber M. Motor learning induces time-dependent plasticity that is observable at the spinal cord level. J Physiol. (2020) 589(10):1943–63. 10.1113/JP278890 32115702

[106] Giboin LS Weiss B Thomas F Gruber M. Neuroplasticity following short-term strength training occurs at supraspinal level and is specific for the trained task. Acta Physiol. (2018) 222(4):e12998. 10.1111/APHA.12998 29144602

[107] Porter JM Beckerman T. Practicing with gradual increases in contextual interference enhances visuomotor learning. Kinesiology. (2016) 48(2):244–50. 10.26582/K.48.2.5

[108] Abdoli B Farsi A Hasan Barani F. Comparing the effects of errorless and errorful and fixed practices on learning of throwing task. Eur J Exp Biol. (2012) 2012(5):1800–6.

[109] Faran Y Osher Y Sofen Y Shalom DB. Errorful and errorless learning in preschoolers: at what age does the errorful advantage appear? Cogn Dev. (2017) 44:150–6. 10.1016/J.COGDEV.2017.10.002

[110] Tailby R Haslam C. An investigation of errorless learning in memory-impaired patients: improving the technique and clarifying theory. Neuropsychologia. (2003) 41(9):1230–40. 10.1016/S0028-3932(03)00036-8 12753962

[111] Blain B Sharot T. Intrinsic reward: potential cognitive and neural mechanisms. Curr Opin Behav Sci. (2021) 39:113–8. 10.1016/j.cobeha.2021.03.008

[112] Kal E Prosée R Winters M Van Der Kamp J. Does implicit motor learning lead to greater automatization of motor skills compared to explicit motor learning? A systematic review. PLoS One. (2018) 13:9. 10.1371/journal.pone.0203591 PMC6124806 30183763

[113] Poolton JM Zachry TL. So you want to learn implicitly? Coaching and learning through implicit motor learning techniques. Int J Sports Sci Coach. (2007) 2(1):67–78. 10.1260/174795407780367177

[114] Cabral DAR Wilson AE Miller MW. The effect of implicit learning on motor performance under psychological pressure: a systematic review and meta-analysis. Sport Exerc Perform Psychol. (2022) 11(3):245–63. 10.1037/SPY0000286

[115] Braun S Kleynen M Van Heel T Kruithof N Wade D Beurskens A. The effects of mental practice in neurological rehabilitation; a systematic review and meta-analysis. Front Hum Neurosci. (2013) 7:7–390. 10.3389/FNHUM.2013.00390 23935572 PMC3731552

[116] Di Nuovo S De La Cruz V Conti D Buono S Di Nuovo A. Mental imagery: rehabilitation through simulation. Life Span Disabil. (2014) 17(1):89–118.

[117] Schuster C Hilfiker R Amft O Scheidhauer A Andrews B Butler J Best practice for motor imagery: a systematic literature review on motor imagery training elements in five different disciplines. BMC Med. (2011) 9(1):75. 10.1186/1741-7015-9-75 21682867 PMC3141540

[118] Wälchli M Ruffieux J Bourquin Y Keller M Taube W. Maximizing performance: augmented feedback, focus of attention, and/or reward? Med Sci Sports Exercise. (2016) 48(4):714–9. 10.1249/MSS.0000000000000818 PMC5638420 26587843

[119] An J Chua LK Wulf G. Optimising golf putting. Int J Sport Exerc Psychol. (2020) 19(5):882–94. 10.1080/1612197X.2020.1854823

[120] Bahrami S Abdoli B Farsi A Aghdaei M Simpson T. The effect of large visual illusion and external focus of attention on gaze behavior and learning of dart throw skill. J Mot Learn Dev. (2022) 10(3):469–84. 10.1123/JMLD.2022-0043

[121] Horbacewicz J. Effect of blocked versus random practice on physical therapy students’ manual force modulation. Percept Mot Skills. (2018) 125(6):1173–85. 10.1177/0031512518797845 30185111

[122] Pesce C Croce R Ben-Soussan TD Vazou S McCullick B Tomporowski PD Variability of practice as an interface between motor and cognitive development. Int J Sport Exerc Psychol. (2016) 17(2):133–52. 10.1080/1612197X.2016.1223421

[123] Porte MC Xeroulis G Reznick RK Dubrowski A. Verbal feedback from an expert is more effective than self-accessed feedback about motion efficiency in learning new surgical skills. Am J Surg. (2007) 193(1):105–10. 10.1016/J.AMJSURG.2006.03.016 17188099

[124] Saraiva M Vilas-Boas JP Fernandes OJ Castro MA. Effects of motor task difficulty on postural control complexity during dual tasks in young adults: a nonlinear approach. Sensors. (2023) 23(2):628. 10.3390/S23020628 36679423 PMC9866022

[125] Sigrist R Rauter G Marchal-Crespo L Riener R Wolf P. Sonification and haptic feedback in addition to visual feedback enhances complex motor task learning. Exp Brain Res. (2014) 233(3):909–25. 10.1007/S00221-014-4167-7 25511166

[126] Sullivan KJ Kantak SS Burtner PA. Motor learning in children: feedback effects on skill acquisition. Phys Ther. (2008) 88(6):720–32. 10.2522/PTJ.20070196 18339797

[127] Zondervan DK Friedman N Chang E Zhao X Augsburger R Reinkensmeyer DJ Home-based hand rehabilitation after chronic stroke: randomized, controlled single-blind trial comparing the music glove with a conventional exercise program. J Rehabil Res Dev. (2016) 53(4):457–72. 10.1682/JRRD.2015.04.0057 27532880

[128] Dumas-Mallet E Button KS Boraud T Gonon F Munafò MR. Low statistical power in biomedical science: a review of three human research domains. R Soc Open Sci. (2017) 4:2. 10.1098/RSOS.160254 PMC5367316 28386409

[129] Broadie M. Assessing golfer performance on the PGA tour. Interfaces (Providence). (2011) 42:2–27. 10.2307/41472743

[130] Brožka M Gryc T Miřátský P Zahálka F. An assessment of the relationships between ball flight results, impact factors, and golf performance. Hum Mov. (2021) 23(1):1–9. 10.5114/HM.2021.104180

[131] Collins D Kamin S. The performance coach. In: Murphy SM, editor. The Oxford Handbook of Sport and Performance Psychology. Oxford Library of Psychology (2012). p. 692–706. 10.1093/OXFORDHB/9780199731763.013.0037

