# Supplementary material for: Motor learning in golf—a systematic review
Source: Front Sports Act Living. 2024 Feb 14;6:1324615. doi: 10.3389/fspor.2024.1324615 (PMC10899359; doi:10.3389/fspor.2024.1324615)
Supplement: Supplementary file 1 [file Datasheet1.docx]

# Appendix: Systematic literature search:

PICO framework:
How do specific motor learning methods (I) affect the performance (O) of participants (P) in a golf-specific task, compared to representative comparison groups (C) that receive no or a different motor learning intervention?

Population: People learning a golf-specific task – search terms: (golf OR putt OR chip OR pitch OR swing OR full swing)
Intervention: motor learning strategies – search terms: (motor learning OR motor control OR cognitive training OR errorful learning OR errorless learning OR contextual interference OR variable practice OR random practice OR blocked practice OR distributed practice OR feedback OR augmented feedback OR knowledge of results OR knowledge of performance OR implicit learning OR explicit learning OR focus of attention OR internal focus OR external focus)
Comparison: no learning or different learning intervention – search-terms: none
Outcome: increased performance – search-terms: (performance OR skill OR acquisition OR skill level OR score OR distance OR accuracy OR precision OR velocity OR speed)

Full search: (golf OR putt OR chip OR pitch OR swing OR full swing) AND (motor learning OR motor control OR cognitive training OR errorful learning OR errorless learning OR contextual interference OR variable practice OR random practice OR blocked practice OR distributed practice OR feedback OR augmented feedback OR knowledge of results OR knowledge of performance OR implicit learning OR explicit learning OR focus of attention OR internal focus OR external focus) AND (performance OR skill acquisition OR skill level OR score OR distance OR accuracy OR precision OR velocity OR speed)

**Risk of Bias Assessment**
